# Supplementary material for: Strong Early Impact of Letrozole on Ovulation Induction Outperforms Clomiphene Citrate in Polycystic Ovary Syndrome
Source: Pharmaceuticals (Basel). 2024 Jul 22;17(7):971. doi: 10.3390/ph17070971 (PMC11280468; doi:10.3390/ph17070971)
Supplement: Supplementary file 1 [file pharmaceuticals-17-00971-s001.zip › pharmaceuticals-3063741-supplementary.pdf]

Supplementary Material

Rita Zsuzsanna Vajna, András Mihály Géczi, Fanni Adél Meznerics, Nándor Ács, Péter Hegyi, Emma Zoé Feig, Péter Fehérvári, Szilvia Kiss-Dala, Szabolcs Várbíró, Judit Réka Hetthessy, Levente Sára

Table S1. Summary of Findings Table

Table S2. PRISMA 2020 [1] Checklist

Figure S1. Forest plot for endometrial thickness (ET) – in ovulating patients only

Figure S2. Forest plot for number of dominant follicles – in all patients

Figure S3. Forest plot for number of dominant follicles – in ovulating patients only

Figure S4. Forest plot for diameter of dominant follicles

Figure S5. Forest plot for pregnancy rate – in ovulating patients only

Figure S6. Forest plot for multiple pregnancy rate

Figure S7. Forest plot for miscarriage rate

Figure S8. Forest plot for monofollicular development rate

Figure S9. Forest plot for multifollicular development rate

Figure S10. Funnel plot for endometrial thickness (ET) – in all patients

Figure S11. Funnel plot for ovulation rate

Figure S12. Funnel plot for pregnancy rate – in all patients

Figure S13. Funnel plot for resistance index (RI) of subendometrial arteries

Figure S14. Funnel plot for pulsatility index (PI) of subendometrial arteries

Figure S15. Risk of bias assessment of the studies included in the meta-analysis assessing endometrial thickness (ET) [2-12] using the revised tool for assessing risk of bias in randomized trials (Rob 2)

Figure S16. Risk of bias assessment of the studies included in the meta-analysis assessing endometrial thickness (ET) [2-12] broken down to tools, shown in percentage

Figure S17. Risk of bias assessment of the studies included in the meta-analysis assessing number of dominant follicles [5-8,10-14] using the revised tool for assessing risk of bias in randomized trials (Rob 2)

Figure S18. Risk of bias assessment of the studies included in the meta-analysis assessing number of dominant follicles [5-8,10-14] broken down to tools, shown in percentage

Figure S19. Risk of bias assessment of the studies included in the meta-analysis assessing diameter of dominant follicles [6,7,10,14] using the revised tool for assessing risk of bias in randomized trials (Rob 2)

Figure S20. Risk of bias assessment of the studies included in the meta-analysis assessing diameter of dominant follicles [6,7,10,14] broken down to tools, shown in percentage

Figure S21. Risk of bias assessment of the studies included in the meta-analysis assessing mono-and multifollicular development [2,3,9] rate using the revised tool for assessing risk of bias in randomized trials (Rob 2)

Figure S22. Risk of bias assessment of the studies included in the meta-analysis assessing mono-and multifollicular development [2,3,9] rate broken down to tools, shown in percentage

Figure S23. Risk of bias assessment of the studies included in the meta-analysis assessing ovulation rate [2-6,10-12,15] using the revised tool for assessing risk of bias in randomized trials (Rob 2)

Figure S24. Risk of bias assessment of the studies included in the meta-analysis assessing ovulation rate [2-6,10-12,15] broken down to tools, shown in percentage

Figure S25. Risk of bias assessment of the studies included in the meta-analysis assessing pregnancy rate [2,5,7,9-16] using the revised tool for assessing risk of bias in randomized trials (Rob 2)

Figure S26. Risk of bias assessment of the studies included in the meta-analysis assessing pregnancy rate [2,5,7,9-16] broken down to tools, shown in percentage

Figure S27. Risk of bias assessment of the studies included in the meta-analysis assessing single and multiple pregnancy rate [5,7-9,11,13,16] using the revised tool for assessing risk of bias in randomized trials (Rob 2)

Figure S28. Risk of bias assessment of the studies included in the meta-analysis assessing single and multiple pregnancy rate [5,7-9,11,13,16] broken down to tools, shown in percentage

Figure S29. Risk of bias assessment of the studies included in the meta-analysis assessing rate of miscarriage [2,7,11,16] using the revised tool for assessing risk of bias in randomized trials (Rob 2)

Figure S30. Risk of bias assessment of the studies included in the meta-analysis assessing rate of miscarriage [2,7,11,16] broken down to tools, shown in percentage

Figure S31. Risk of bias assessment of the studies included in the meta-analysis assessing resistance index (RI) of subendometrial arteries [5,6,14] using the revised tool for assessing risk of bias in randomized trials (Rob 2)

Figure S32. Risk of bias assessment of the studies included in the meta-analysis assessing resistance index (RI) of subendometrial arteries [5,6,14] broken down to tools, shown in percentage

Figure S33. Risk of bias assessment of the studies included in the meta-analysis assessing pulsatility index (PI) of subendometrial arteries [5,6,14] using the revised tool for assessing risk of bias in randomized trials (Rob 2)

Figure S34. Risk of bias assessment of the studies included in the meta-analysis assessing pulsatility index (PI) of subendometrial arteries [5,6,14] broken down to tools, shown in percentage

Figure S35. Risk of bias assessment of the studies included in the systematic review assessing rate of endometrial thickness (ET) [14,15,17-25] using the revised tool for assessing risk of bias in randomized trials (Rob 2)

Figure S36. Risk of bias assessment of the studies included in the systematic review assessing rate of endometrial thickness (ET) [14,15,17-25] broken down to tools, shown in percentage

Figure S37. Risk of bias assessment of the studies included in the systematic review assessing endometrial volume (EV) [6,10] using the revised tool for assessing risk of bias in randomized trials (Rob 2)

Figure S38. Risk of bias assessment of the studies included in the systematic review assessing endometrial volume (EV) [6,10] broken down to tools, shown in percentage

Figure S39. Risk of bias assessment of the studies included in the systematic review assessing endometrial pattern and/or echogenicity [6,15,17] using the revised tool for assessing risk of bias in randomized trials (Rob 2)

Figure S40. Risk of bias assessment of the studies included in the systematic review assessing endometrial pattern and/or echogenicity [6,15,17] broken down to tools, shown in percentage

Figure S41. Risk of bias assessment of the studies included in the systematic review assessing rate of number of dominant follicles [18-24] using the revised tool for assessing risk of bias in randomized trials (Rob 2)

Figure S42. Risk of bias assessment of the studies included in the systematic review assessing rate of number of dominant follicles [18-24] broken down to tools, shown in percentage

Figure S43. Risk of bias assessment of the studies included in the systematic review assessing rate of diameter of dominant follicles [23,24] using the revised tool for assessing risk of bias in randomized trials (Rob 2)

Figure S44. Risk of bias assessment of the studies included in the systematic review assessing rate of diameter of dominant follicles [23,24] broken down to tools, shown in percentage

Figure S45. Risk of bias assessment of the studies included in the systematic review assessing monofollicular development cycles [24,25] using the revised tool for assessing risk of bias in randomized trials (Rob 2)

Figure S46. Risk of bias assessment of the studies included in the systematic review assessing monofollicular development cycles [24,25] broken down to tools, shown in percentage

Figure S47. Risk of bias assessment of the studies included in the systematic review assessing ovulation rate [17,20-22,25,26] using the revised tool for assessing risk of bias in randomized trials (Rob 2).

Figure S48. Risk of bias assessment of the studies included in the systematic review assessing ovulation rate [17,20-22,25,26] broken down to tools, shown in percentage

Figure S49. Risk of bias assessment of the studies included in the systematic review assessing pregnancy rate [17-26] using the revised tool for assessing risk of bias in randomized trials (Rob 2)

Figure S50. Risk of bias assessment of the studies included in the systematic review assessing pregnancy rate [17-26] broken down to tools, shown in percentage

Figure S51. Risk of bias assessment of the studies included in the systematic review assessing number of multiple pregnancies [18-21,26] using the revised tool for assessing risk of bias in randomized trials (Rob 2)

Figure S52. Risk of bias assessment of the studies included in the systematic review assessing number of multiple pregnancies [18-21,26] broken down to tools, shown in percentage

Figure S53. Risk of bias assessment of the studies included in the systematic review assessing number of miscarriages [19-21,23,24,26] using the revised tool for assessing risk of bias in randomized trials (Rob 2)

Figure S54. Risk of bias assessment of the studies included in the systematic review assessing number of miscarriages [19-21,23,24,26] broken down to tools, shown in percentage

Figure S55. Risk of bias assessment of the studies included in the systematic review assessing live birth rate [11,17,19-21,26] using the revised tool for assessing risk of bias in randomized trials (Rob 2)

Figure S56. Risk of bias assessment of the studies included in the systematic review assessing live birth rate [11,17,19-21,26] broken down to tools, shown in percentage

Figure S57. Risk of bias assessment of the studies included in the systematic review assessing number of ectopic pregnancies [16] using the revised tool for assessing risk of bias in randomized trials (Rob 2)

Figure S58. Risk of bias assessment of the studies included in the systematic review assessing number of ectopic pregnancies [16] broken down to tools, shown in percentage

Figure S59. Risk of bias assessment of the studies included in the systematic review assessing number of fetal anomalies [11,17,21,24,26] using the revised tool for assessing risk of bias in randomized trials (Rob 2)

Figure S60. Risk of bias assessment of the studies included in the systematic review assessing number of fetal anomalies [11,17,21,24,26] broken down to tools, shown in percentage

Figure S61. Risk of bias assessment of the studies included in the systematic review assessing endometrial vascularization index (VI), flow index (FI), vascularization flow index (VFI) and detection rate of endometrial-subendometrial blood flow [5,6,10] using the revised tool for assessing risk of bias in randomized trials (Rob 2)

Figure S62. Risk of bias assessment of the studies included in the systematic review assessing endometrial vascularization index (VI), flow index (FI), vascularization flow index (VFI) detection rate of endometrial-subendometrial blood flow [5,6,10] broken down to tools, shown in percentage

Figure S63. Risk of bias assessment of the studies included in the systematic review assessing systolic velocity (SV)/diastolic velocity (DV) of subendometrial arteries [14] using the revised tool for assessing risk of bias in randomized trials (Rob 2)

Figure S64. Risk of bias assessment of the studies included in the systematic review assessing systolic velocity (SV)/diastolic velocity (DV) of subendometrial arteries [14]broken down to tools, shown in percentage

Figure S65. Risk of bias assessment of the studies included in the systematic review assessing resistance index (RI) and pulsatility index (PI) of uterine arteries [6,10] using the revised tool for assessing risk of bias in randomized trials (Rob 2)

Figure S66. Risk of bias assessment of the studies included in the systematic review assessing resistance index (RI) and pulsatility index (PI) of uterine arteries [6,10] broken down to tools, shown in percentage.

Figure S67. Risk of bias assessment of the studies included in the systematic review assessing vascular endothelial growth factor (VEGF) and/or integrin alpha vß3 [10,14] using the revised tool for assessing risk of bias in randomized trials (Rob 2)

Figure S68. Risk of bias assessment of the studies included in the systematic review assessing vascular endothelial growth factor (VEGF) and/or integrin alpha vß3 [10,14] broken down to tools, shown in percentage

Figure S69. Risk of bias assessment of the studies included in the systematic review assessing resistance index (RI) and pulsatility index (PI) of subendometrial arteries [18] using the revised tool for assessing risk of bias in randomized trials (Rob 2)

Figure S70. Risk of bias assessment of the studies included in the systematic review assessing resistance index (RI) and pulsatility index (PI) of subendometrial arteries [18] broken down to tools, shown in percentage

Supplementary References

Table S1. Summary of Findings Table [27].

Certainty assessment							№ of patients		Effect		Certainty	Importance
№ of studies	Study design	Risk of bias	Inconsistency	Indirectness	Imprecision	Other considerations	Letrozole for ovulation induction	Clomiphene citrate for ovulation induction	Relative (95% CI)	Absolute (95% CI)		
**Endometrial thickness (ET) (follow-up: mean 1 months)**												
11	randomised trials	serious[a,b]	not serious	not serious	not serious[c]	none	828	823	-	MD **1.7 mm higher** (0.55 higher to 2.86 higher)	⨁⨁⨁◯ Moderate	IMPORTANT
**Number of dominant follicles (follow-up: mean 1 months)**												
9	randomised trials	serious[d]	not serious	not serious	not serious[c]	none	631	633	-	MD **0.4 lower** (0.84 lower to 0.03 higher)	⨁⨁⨁◯ Moderate	IMPORTANT

Certainty assessment							№ of patients		Effect		Certainty	Importance
**№ of studies**	**Study design**	**Risk of bias**	**Inconsistency**	**Indirectness**	**Imprecision**	**Other considerations**	**Letrozole for ovulation induction**	**Clomiphene citrate for ovulation induction**	**Relative (95% CI)**	**Absolute (95% CI)**		
**Diameter of dominant follicles (follow-up: mean 1 months)**												
4	randomised trials	serious[d]	not serious	not serious	not serious[c]	none	330	330	-	MD **0.58 mm higher** (0.17 lower to 1.32 higher)	⊕⊕⊕◯ Moderate	IMPORTANT
**Ovulation rate (follow-up: 1 months)**												
9	randomised trials	serious[a,b,d]	not serious	not serious	not serious[c]	none	554/754 (73.5%)	478/756 (63.2%)	**OR 1.80** (1.21 to 2.69)	**124 more per 1 000** (from 43 more to 190 more)	⊕⊕⊕◯ Moderate	CRITICAL
**Pregnancy rate (follow-up: 1 months)**												

Certainty assessment							№ of patients		Effect		Certainty	Importance
№ of studies	Study design	Risk of bias	Inconsistency	Indirectness	Imprecision	Other considerations	Letrozole for ovulation induction	Clomiphene citrate for ovulation induction	Relative (95% CI)	Absolute (95% CI)		
11	randomised trials	serious[d]	not serious	not serious	not serious[c]	none	254/704 (36.1%)	165/706 (23.4%)	**OR 1.96** (1.37 to 2.81)	**140 more per 1 000** (from 61 more to 228 more)	⊕⊕⊕◯ Moderate	CRITICAL
**Number of single pregnancies (follow-up: 1 months)**												
8	randomised trials	serious[a,b]	not serious	not serious	not serious[c]	none	153/157 (97.5%)	91/96 (94.8%)	**OR 2.46** (0.74 to 8.18)	**30 more per 1 000** (from 17 fewer to 45 more)	⊕⊕⊕◯ Moderate	CRITICAL
**Number of multiple pregnancies (follow-up: 1 months)**												

Certainty assessment							№ of patients		Effect		Certainty	Importance
№ of studies	Study design	Risk of bias	Inconsistency	Indirectness	Imprecision	Other considerations	Letrozole for ovulation induction	Clomiphene citrate for ovulation induction	Relative (95% CI)	Absolute (95% CI)		
8	randomised trials	serious[a,b]	not serious	not serious	not serious[c]	none	4/157 (2.5%)	5/96 (5.2%)	**OR 0.41** (0.12 to 1.35)	**30 fewer per 1 000** (from 46 fewer to 17 more)	⊕⊕⊕◯ Moderate	CRITICAL
**Number of miscarriage (follow-up: 1 months)**												
4	randomised trials	serious[a,b]	not serious	not serious	not serious[c]	none	11/98 (11.2%)	11/62 (17.7%)	**OR 0.62** (0.19 to 1.98)	**59 fewer per 1 000** (from 138 fewer to 122 more)	⊕⊕⊕◯ Moderate	CRITICAL
**Resistance index (RI) of subendometrial arteries (follow-up: mean 1 months)**												

Certainty assessment							№ of patients		Effect		Certainty	Importance
№ of studies	Study design	Risk of bias	Inconsistency	Indirectness	Imprecision	Other considerations	Letrozole for ovulation induction	Clomiphene citrate for ovulation induction	Relative (95% CI)	Absolute (95% CI)		
3	randomised trials	serious[d]	not serious	not serious	not serious[c]	none	230	230	-	MD **0.15 lower** (0.27 lower to 0.04 lower)	⊕⊕⊕◯ Moderate	IMPORTANT
**Pulsatility index (PI) of subendometrial arteries (follow-up: mean 1 months)**												
3	randomised trials	serious[d]	not serious	not serious	not serious[c]	none	230	230	-	MD **0.17 lower** (0.81 lower to 0.47 higher)	⊕⊕⊕◯ Moderate	IMPORTANT
**Monofollicular development (follow-up: 1 months)**												

Certainty assessment							№ of patients		Effect		Certainty	Importance
№ of studies	Study design	Risk of bias	Inconsistency	Indirectness	Imprecision	Other considerations	Letrozole for ovulation induction	Clomiphene citrate for ovulation induction	Relative (95% CI)	Absolute (95% CI)		
3	randomised trials	serious[a,b]	not serious	not serious	not serious[c]	none	175/282 (62.1%)	137/281 (48.8%)	**OR 1.99** (0.62 to 6.34)	**167 more per 1 000** (from 117 fewer to 370 more)	⊕⊕⊕◯ Moderate	IMPORTANT
**Multifollicular developlent (follow-up: 1 months)**												
3	randomised trials	serious[a,b]	not serious	not serious	not serious[c]	none	107/282 (37.9%)	144/281 (51.2%)	**OR 0.50** (0.16 to 1.61)	**168 fewer per 1 000** (from 368 fewer to 116 more)	⊕⊕⊕◯ Moderate	IMPORTANT

CI: confidence interval; MD: mean difference; OR: odds ratio

Explanations:

a. We found some articles, in wich we didn't find informations about the allocation sequence concelated until participants were enrolled and assigned to interventions.

b. We didn't find informations about were data produced this result analysed in accordance with a prespecified analysis plan that was finalized before unblinded outcome date were available for analysis.

c. Low patient number

d. In some articles the data for this outcome not available for all, or nearly all, participants were randomised, therefore not evidence that the result was not biased by missing outcome data.

Table S2. PRISMA 2020 [1] Checklist

Section and Topic	Item #	Checklist item	Location where item is reported
**TITLE**			
Title	1	Identify the report as a systematic review.	1
**ABSTRACT**			
Abstract	2	See the PRISMA 2020 for Abstracts checklist.	1
**INTRODUCTION**			
Rationale	3	Describe the rationale for the review in the context of existing knowledge.	1,2
Objectives	4	Provide an explicit statement of the objective(s) or question(s) the review addresses.	2
**METHODS**			
Eligibility criteria	5	Specify the inclusion and exclusion criteria for the review and how studies were grouped for the syntheses.	20,21
Information sources	6	Specify all databases, registers, websites, organisations, reference lists and other sources searched or consulted to identify studies. Specify the date when each source was last searched or consulted.	20
Search strategy	7	Present the full search strategies for all databases, registers and websites, including any filters and limits used.	20
Selection process	8	Specify the methods used to decide whether a study met the inclusion criteria of the review, including how many reviewers screened each record and each report retrieved, whether they worked independently, and if applicable, details of automation tools used in the process.	21
Data collection process	9	Specify the methods used to collect data from reports, including how many reviewers collected data from each report, whether they worked independently, any processes for obtaining or confirming data from study investigators, and if applicable, details of automation tools used in the process.	21
Data items	10a	List and define all outcomes for which data were sought. Specify whether all results that were compatible with each outcome domain in each study were sought (e.g. for all measures, time points, analyses), and if not, the methods used to decide which results to collect.	21
	10b	List and define all other variables for which data were sought (e.g. participant and intervention characteristics, funding sources). Describe any assumptions made about any missing or unclear information.	21
Study risk of bias assessment	11	Specify the methods used to assess risk of bias in the included studies, including details of the tool(s) used, how many reviewers assessed each study and whether they worked independently, and if applicable, details of automation tools used in the process.	21,23

Section and Topic	Item #	Checklist item	Location where item is reported
Effect measures	12	Specify for each outcome the effect measure(s) (e.g. risk ratio, mean difference) used in the synthesis or presentation of results.	22,23

Section and Topic	Item #	Checklist item	Location where item is reported
Synthesis methods	13a	Describe the processes used to decide which studies were eligible for each synthesis (e.g. tabulating the study intervention characteristics and comparing against the planned groups for each synthesis (item #5)).	22,23
	13b	Describe any methods required to prepare the data for presentation or synthesis, such as handling of missing summary statistics, or data conversions.	22,23
	13c	Describe any methods used to tabulate or visually display results of individual studies and syntheses.	22,23
	13d	Describe any methods used to synthesize results and provide a rationale for the choice(s). If meta-analysis was performed, describe the model(s), method(s) to identify the presence and extent of statistical heterogeneity, and software package(s) used.	22,23
	13e	Describe any methods used to explore possible causes of heterogeneity among study results (e.g. subgroup analysis, meta-regression).	22,23
	13f	Describe any sensitivity analyses conducted to assess robustness of the synthesized results.	22,23
Reporting bias assessment	14	Describe any methods used to assess risk of bias due to missing results in a synthesis (arising from reporting biases).	22,23
Certainty assessment	15	Describe any methods used to assess certainty (or confidence) in the body of evidence for an outcome.	22,23
**RESULTS**			
Study selection	16a	Describe the results of the search and selection process, from the number of records identified in the search to the number of studies included in the review, ideally using a flow diagram.	2,3, Figure 1.
	16b	Cite studies that might appear to meet the inclusion criteria, but which were excluded, and explain why they were excluded.	Figure 1.
Study characteristics	17	Cite each included study and present its characteristics.	Table 1A and B.
Risk of bias in studies	18	Present assessments of risk of bias for each included study.	Figure S15-Figure S70.
Results of individual studies	19	For all outcomes, present, for each study: (a) summary statistics for each group (where appropriate) and (b) an effect estimate and its precision (e.g. confidence/credible interval), ideally using structured tables or plots.	8-10, Figure 2., Figure 3., Table 2 A and B., Figure S1-Figure S14.
Results of	20a	For each synthesis, briefly summarise the characteristics and	Table S1.

Section and Topic	Item #	Checklist item	Location where item is reported
syntheses		risk of bias among contributing studies.	
	20b	Present results of all statistical syntheses conducted. If meta-analysis was done, present for each the summary estimate and its precision (e.g. confidence/credible interval) and measures of statistical heterogeneity. If comparing groups, describe the direction of the effect.	8-10 Figure 2., Figure 3., Figure S1-Figure S14.
	20c	Present results of all investigations of possible causes of heterogeneity among study results.	22,23, Figure 2., Figure 3., Figure S1-Figure S14.
	20d	Present results of all sensitivity analyses conducted to assess the robustness of the synthesized results.	Table S1., Figure S10-Figure S70.
Reporting biases	21	Present assessments of risk of bias due to missing results (arising from reporting biases) for each synthesis assessed.	21,23, Figure S10-Figure S14.
Certainty of evidence	22	Present assessments of certainty (or confidence) in the body of evidence for each outcome assessed.	Table S1.
**DISCUSSION**			
Discussion	23a	Provide a general interpretation of the results in the context of other evidence.	18-20
	23b	Discuss any limitations of the evidence included in the review.	23
	23c	Discuss any limitations of the review processes used.	23
	23d	Discuss implications of the results for practice, policy, and future research.	23
**OTHER INFORMATION**			
Registration and protocol	24a	Provide registration information for the review, including register name and registration number, or state that the review was not registered.	1,20
	24b	Indicate where the review protocol can be accessed, or state that a protocol was not prepared.	1,20
	24c	Describe and explain any amendments to information provided at registration or in the protocol.	1,20
Support	25	Describe sources of financial or non-financial support for the review, and the role of the funders or sponsors in the review.	24
Competing interests	26	Declare any competing interests of review authors.	24
Availability of data, code and other materials	27	Report which of the following are publicly available and where they can be found: template data collection forms; data extracted from included studies; data used for all analyses; analytic code; any other materials used in the review.	Table 1A and B, Table 2 A and B, Table S1.

Section and Topic	Item #	Checklist item	Reported (Yes/No)
**TITLE**			
Title	1	Identify the report as a systematic review.	Yes
**BACKGROUND**			
Objectives	2	Provide an explicit statement of the main objective(s) or question(s) the review addresses.	Yes
**METHODS**			
Eligibility criteria	3	Specify the inclusion and exclusion criteria for the review.	Yes
Information sources	4	Specify the information sources (e.g. databases, registers) used to identify studies and the date when each was last searched.	Yes
Risk of bias	5	Specify the methods used to assess risk of bias in the included studies.	No
Synthesis of results	6	Specify the methods used to present and synthesise results.	Yes
**RESULTS**			
Included studies	7	Give the total number of included studies and participants and summarise relevant characteristics of studies.	Yes
Synthesis of results	8	Present results for main outcomes, preferably indicating the number of included studies and participants for each. If meta-analysis was done, report the summary estimate and confidence/credible interval. If comparing groups, indicate the direction of the effect (i.e. which group is favoured).	Yes
**DISCUSSION**			
Limitations of evidence	9	Provide a brief summary of the limitations of the evidence included in the review (e.g. study risk of bias, inconsistency and imprecision).	No
Interpretation	10	Provide a general interpretation of the results and important implications.	Yes
**OTHER**			
Funding	11	Specify the primary source of funding for the review.	No
Registration	12	Provide the register name and registration number.	Yes

Figure S1. Forest plot for endometrial thickness (ET) – in ovulating patients only

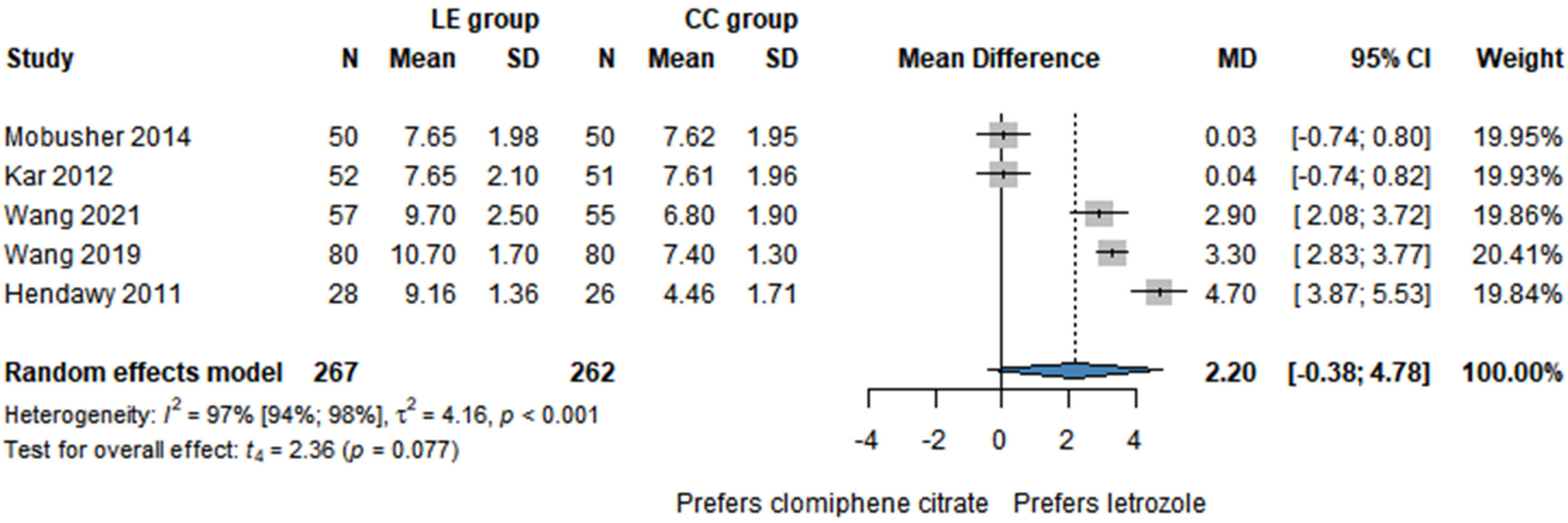

LE-letrozole; CC-clomiphene citrate; N-number of patients; MD-mean difference; SD-standard deviation; CI-confidence interval; p-p-value

Figure S2. Forest plot for number of dominant follicles – in all patients

Study	LE group N	LE group Mean	LE group SD	CC group N	CC group Mean	CC group SD	MD	95% CI	Weight
Hendawy 2011	28	1.20	0.90	26	2.90	1.77	-1.70	[-2.46; -0.94]	7.78%
Atay 2006	51	1.20	0.41	55	2.40	1.15	-1.20	[-1.52; -0.88]	11.15%
Al-Obaidi 2019	40	1.83	0.38	40	2.35	0.92	-0.52	[-0.83; -0.21]	11.26%
Wang 2019	80	1.40	0.70	80	1.80	0.90	-0.40	[-0.65; -0.15]	11.61%
Elsedeek 2011	62	1.23	0.50	62	1.50	0.30	-0.27	[-0.42; -0.12]	12.09%
Wang 2021	90	1.40	0.50	90	1.50	0.60	-0.10	[-0.26; 0.06]	12.03%
Dehbashi 2009	50	1.06	0.95	50	1.14	1.17	-0.08	[-0.50; 0.34]	10.48%
Selim 2012	110	1.36	1.00	110	1.27	1.10	0.09	[-0.19; 0.37]	11.45%
Najafi 2020	120	1.40	0.60	120	1.30	0.40	0.10	[-0.03; 0.23]	12.14%
**Random effects model**	**631**			**633**			**-0.40**	**[-0.84; 0.03]**	**100.00%**
**Prediction interval**								**[-1.67; 0.86]**	

Heterogeneity: $I^2 = 91\%$ [85%; 94%], $\tau^2 = 0.25$, $p < 0.001$
Test for overall effect: $t_8 = -2.12$ ($p = 0.066$)

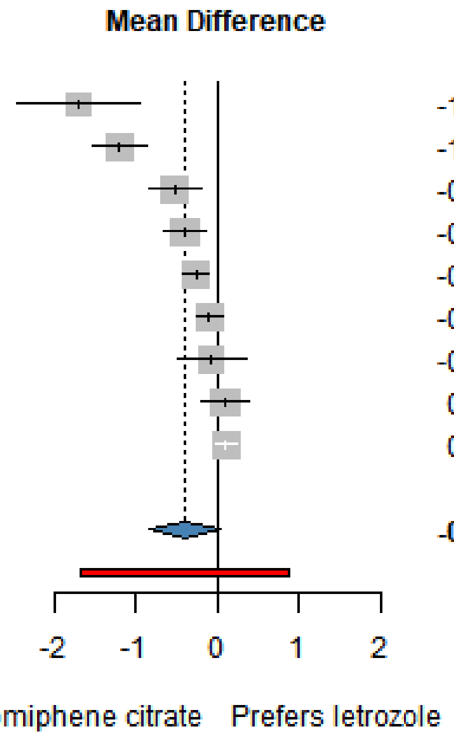

LE-letrozole; CC-clomiphene citrate; N-number of patients; MD-mean difference; SD-standard deviation; CI-confidence interval; p-p-value

Figure S3. Forest plot for number of dominant follicles – in ovulating patients only

Study	LE group N	LE group Mean	LE group SD	CC group N	CC group Mean	CC group SD	MD	95% CI	Weight
Hendawy 2011	28	1.20	0.90	26	2.90	1.77	-1.70	[-2.46; -0.94]	27.19%
Al-Obaidi 2019	40	1.83	0.38	40	2.35	0.92	-0.52	[-0.83; -0.21]	36.00%
Wang 2019	80	1.40	0.70	80	1.80	0.90	-0.40	[-0.65; -0.15]	36.81%
**Random effects model**	**148**			**146**			**-0.80**	**[-2.48; 0.89]**	**100.00%**

Heterogeneity: $I^2 = 80\%$ [38%; 94%], $\tau^2 = 0.36$, $p = 0.006$

Test for overall effect: $t_2 = -2.03$ ($p = 0.179$)

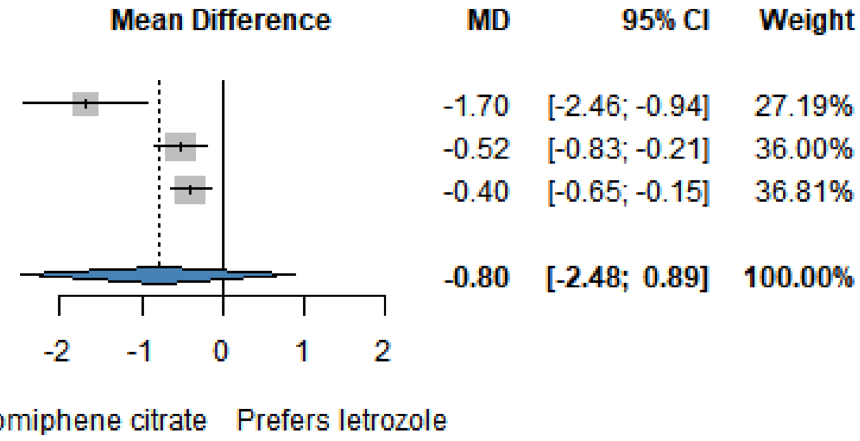

LE-letrozole; CC-clomiphene citrate; N-number of patients; MD-mean difference; SD-standard deviation; CI-confidence interval; p-p-value

Figure S4. Forest plot for diameter of dominant follicles

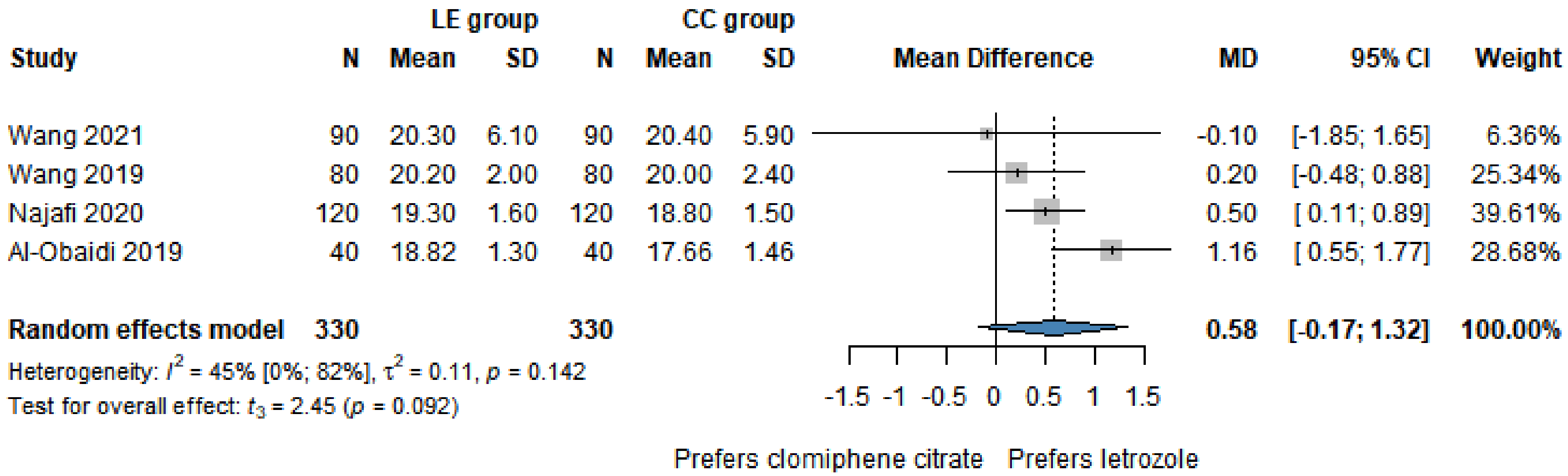

LE-letrozole; CC-clomiphene citrate; N-number of patients; MD-mean difference; SD-standard deviation; CI-confidence interva; p-p-value

Figure S5. Forest plot for pregnancy rate – in ovulating patients only

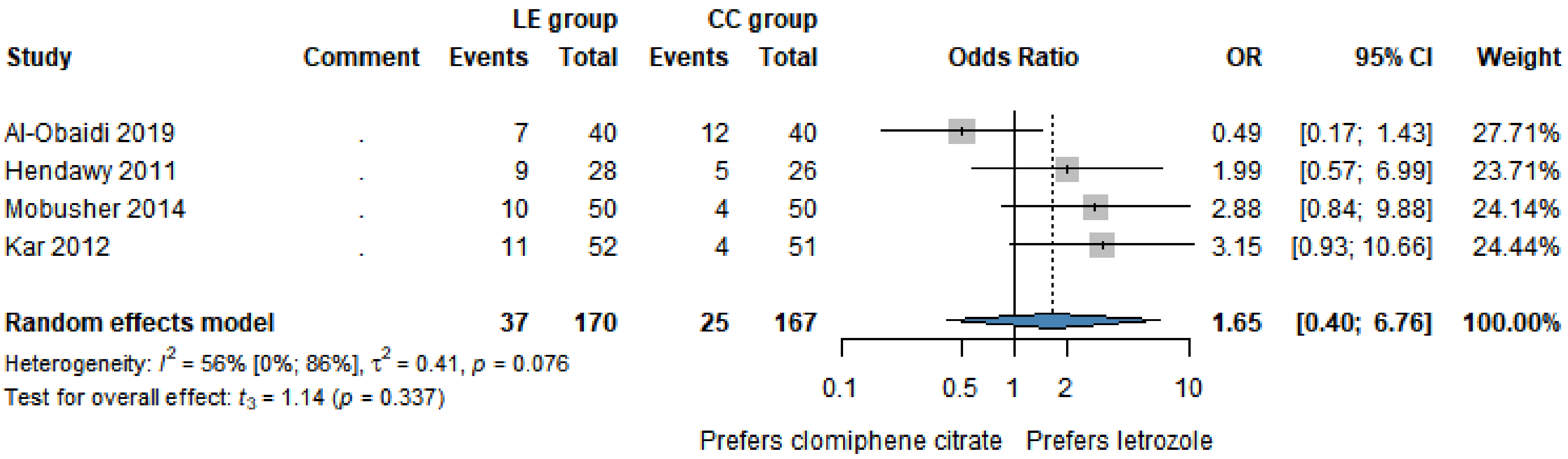

LE-letrozole; CC-clomiphene citrate; N-number of patients; OR-odds ratio; CI-confidence interval; p-p-value

Figure S6. Forest plot for multiple pregnancy rate

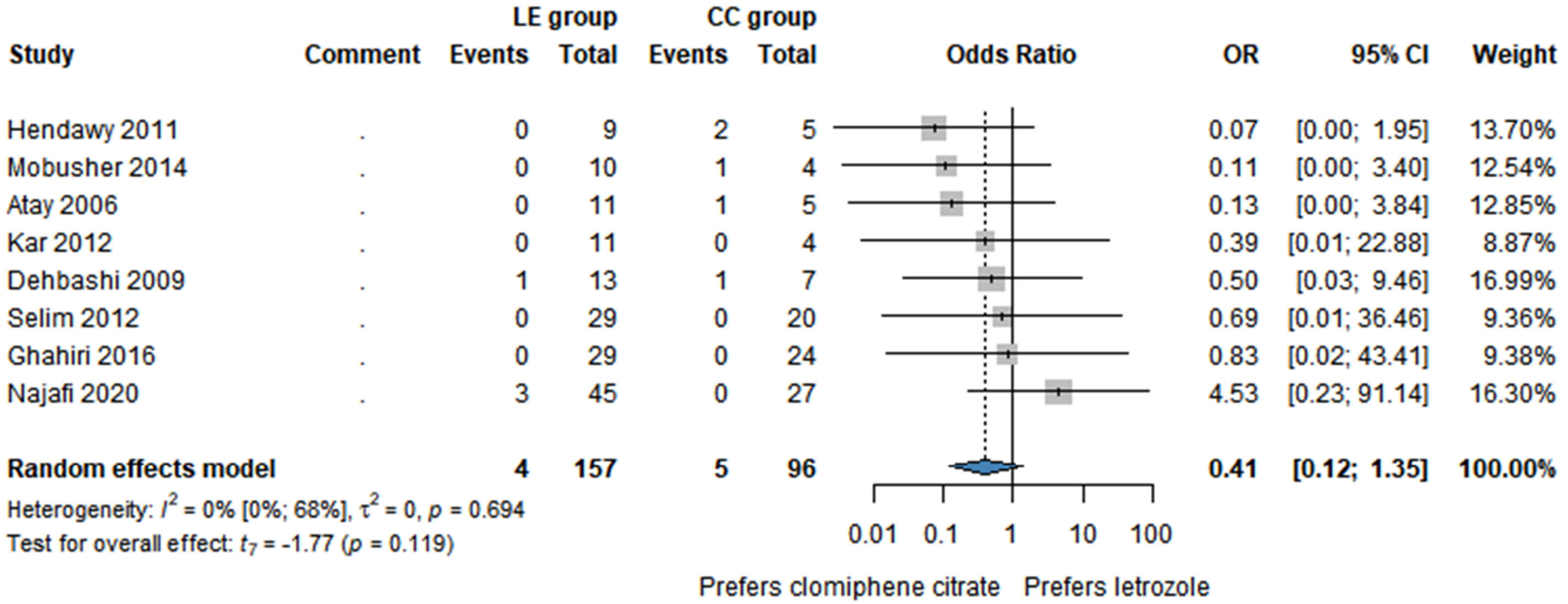

LE-letrozole; CC-clomiphene citrate; N-number of patients; OR-odds ratio; CI-confidence interval; p-p-value

Figure S7. Forest plot for miscarriage rate

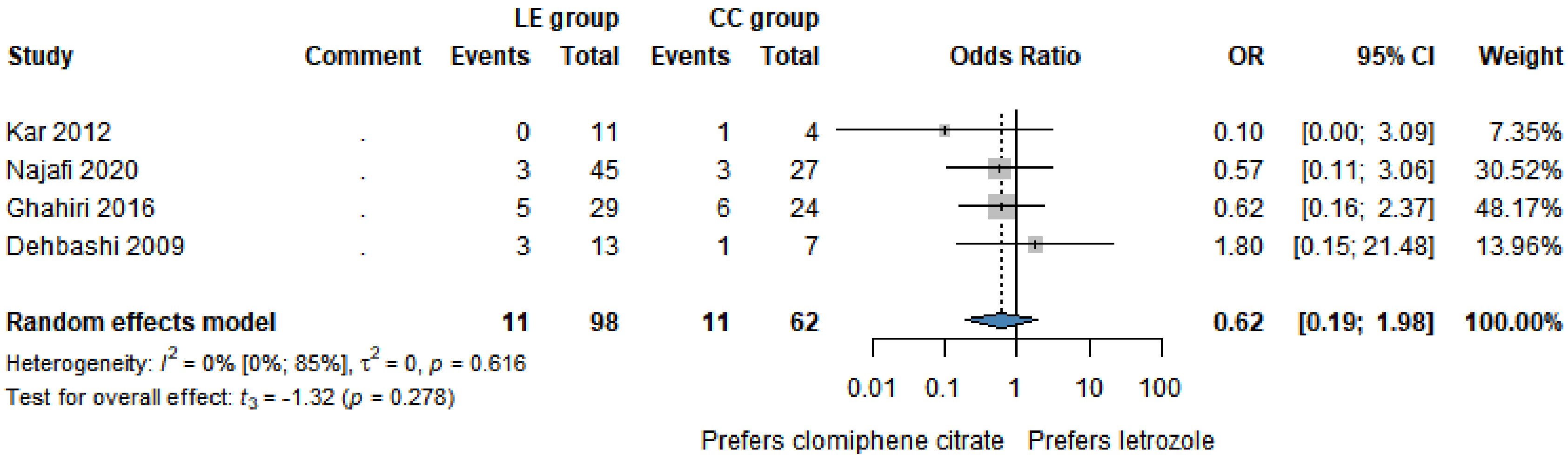

LE-letrozole; CC-clomiphene citrate; N-number of patients; OR-odds ratio; CI-confidence interval; p-p-value

Figure S8. Forest plot for monofollicular development rate

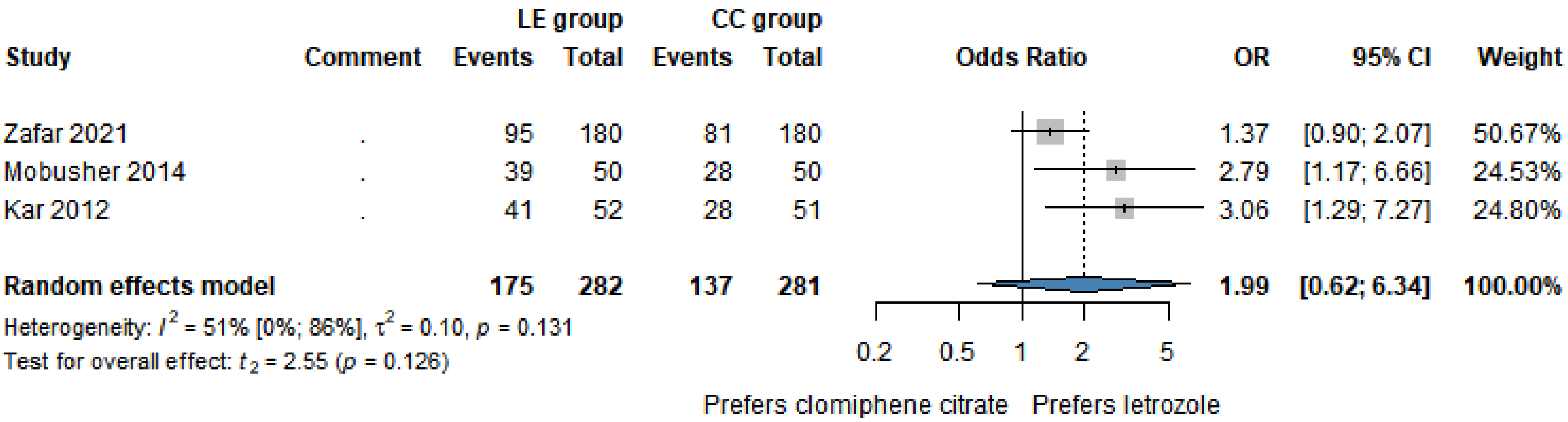

LE-letrozole; CC-clomiphene citrate; N-number of patients; OR-odds ratio; CI-confidence interval; p-p-value

Figure S9. Forest plot for multifollicular development rate

Study	Comment	LE group Events	LE group Total	CC group Events	CC group Total	OR	95% CI	Weight
Kar 2012	.	11	52	23	51	0.33	[0.14; 0.78]	24.80%
Mobusher 2014	.	11	50	22	50	0.36	[0.15; 0.86]	24.53%
Zafar 2021	.	85	180	99	180	0.73	[0.48; 1.11]	50.67%
**Random effects model**		**107**	**282**	**144**	**281**	**0.50**	**[0.16; 1.61]**	**100.00%**

Heterogeneity: $I^2 = 51\%$ [0%; 86%], $\tau^2 = 0.10$, $p = 0.131$

Test for overall effect: $t_2 = -2.55$ ($p = 0.126$)

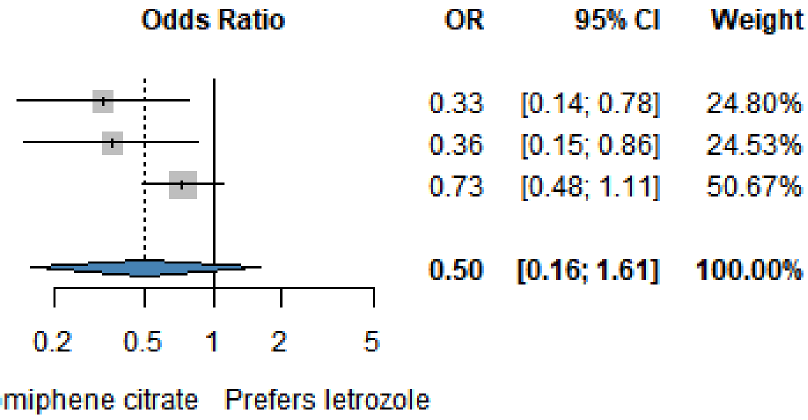

LE-letrozole; CC-clomiphene citrate; N-number of patients; OR-odds ratio; CI-confidence interval; p-p-value

Figure S10. Funnel plot for endometrial thickness (ET) – in all patients

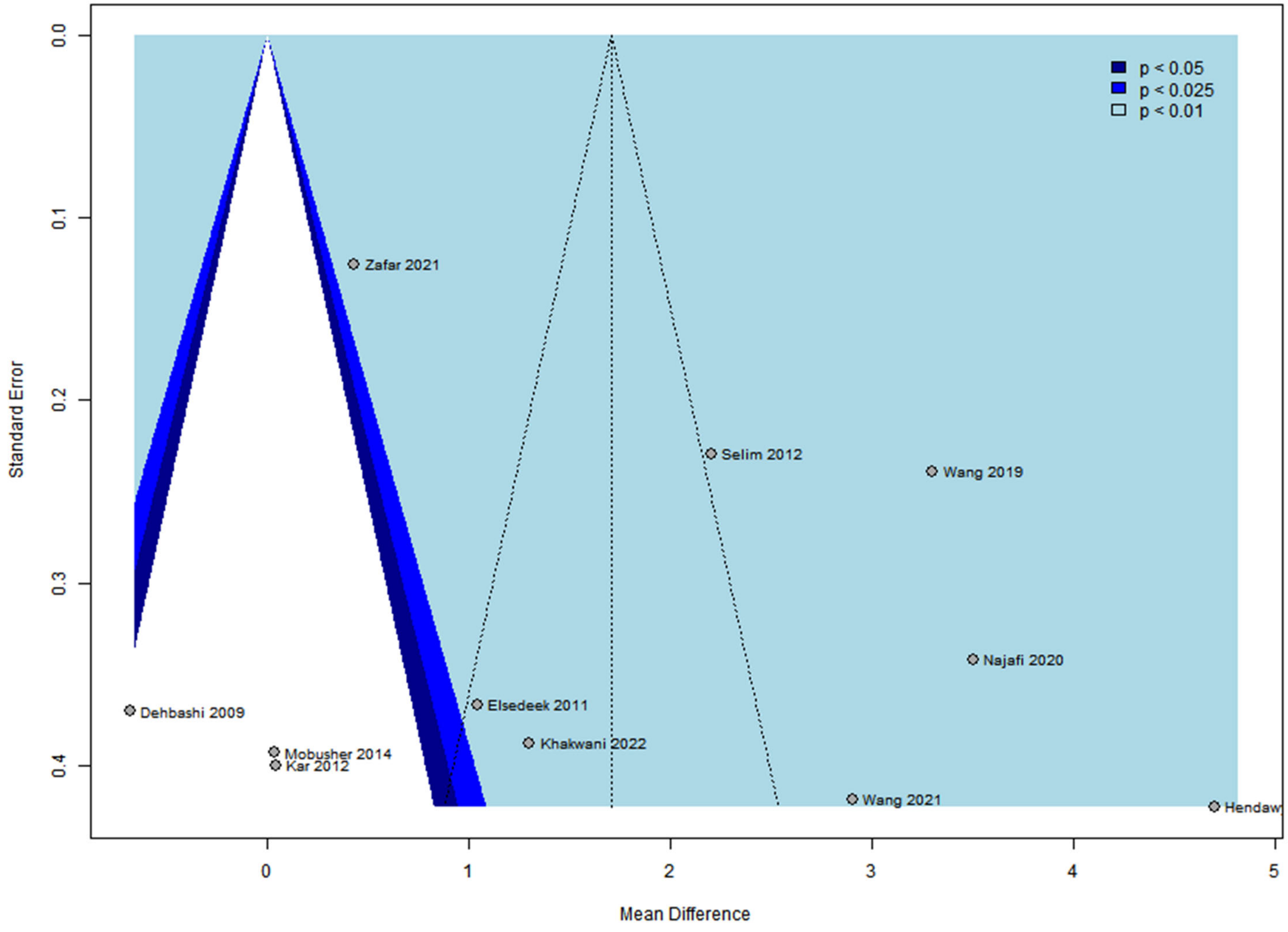

Figure S11. Funnel plot for ovulation rate

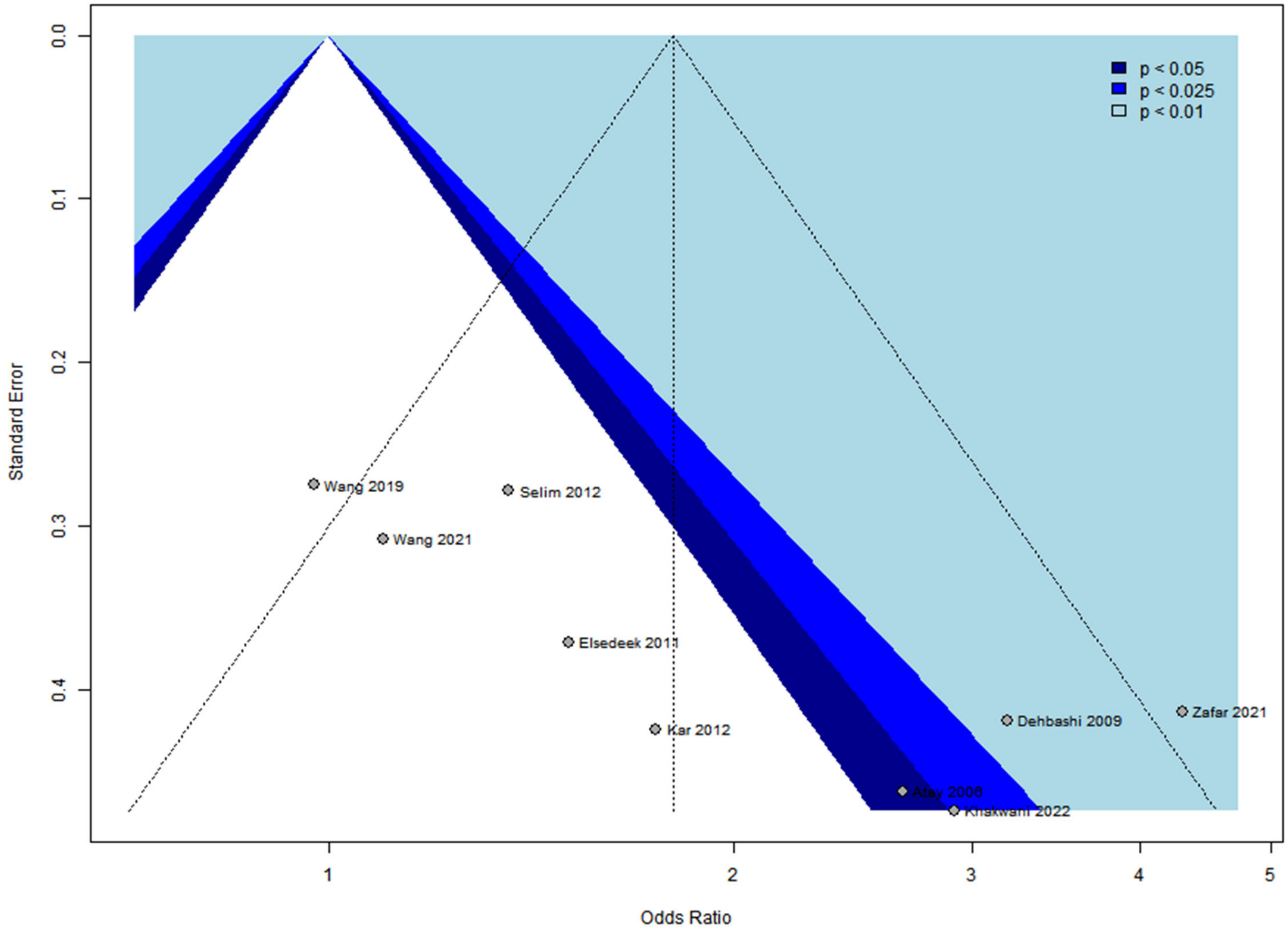

Figure S12. Funnel plot for pregnancy rate – in all patients

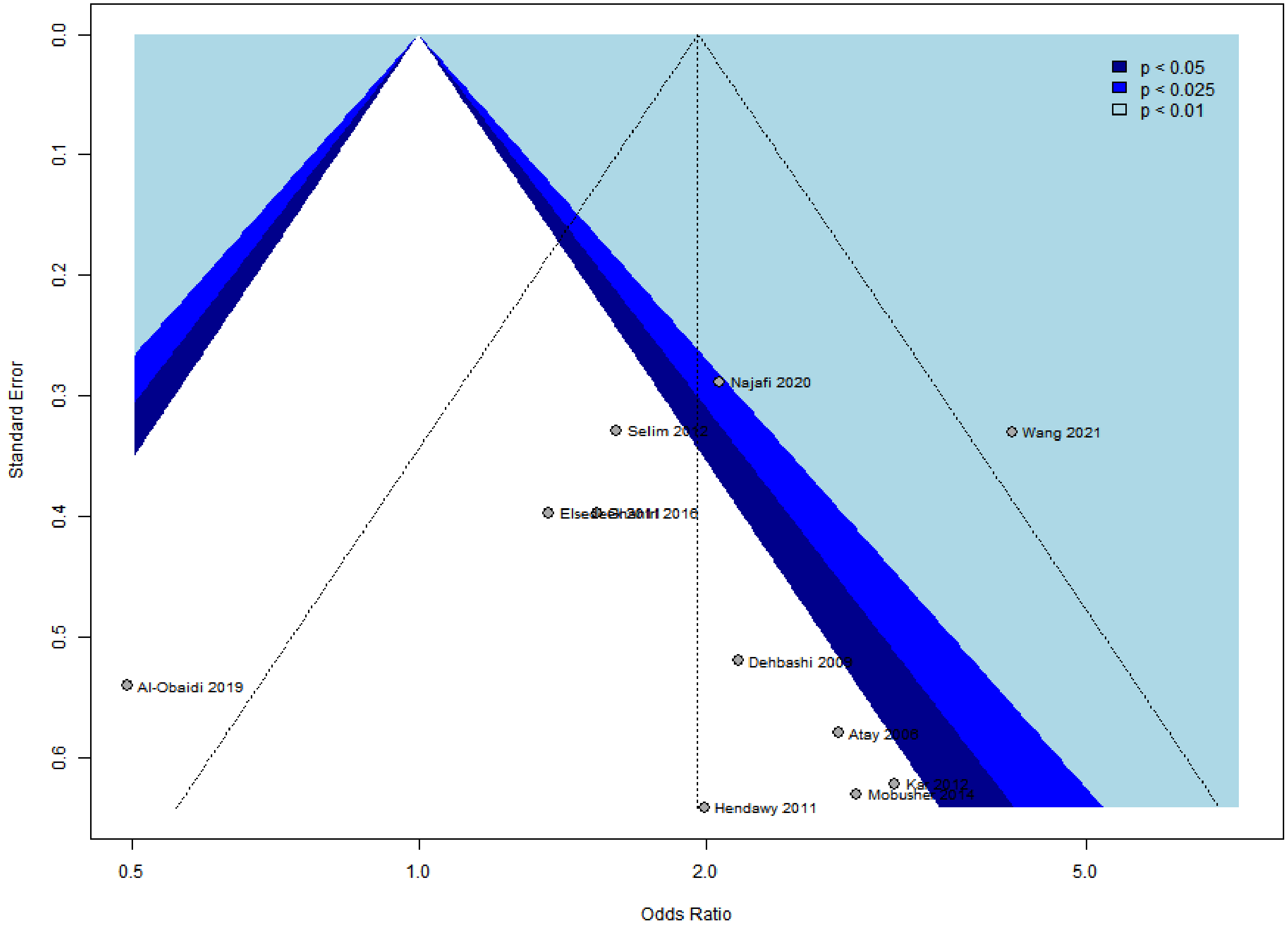

Figure S13. Funnel plot for resistance index (RI) of subendometrial arteries

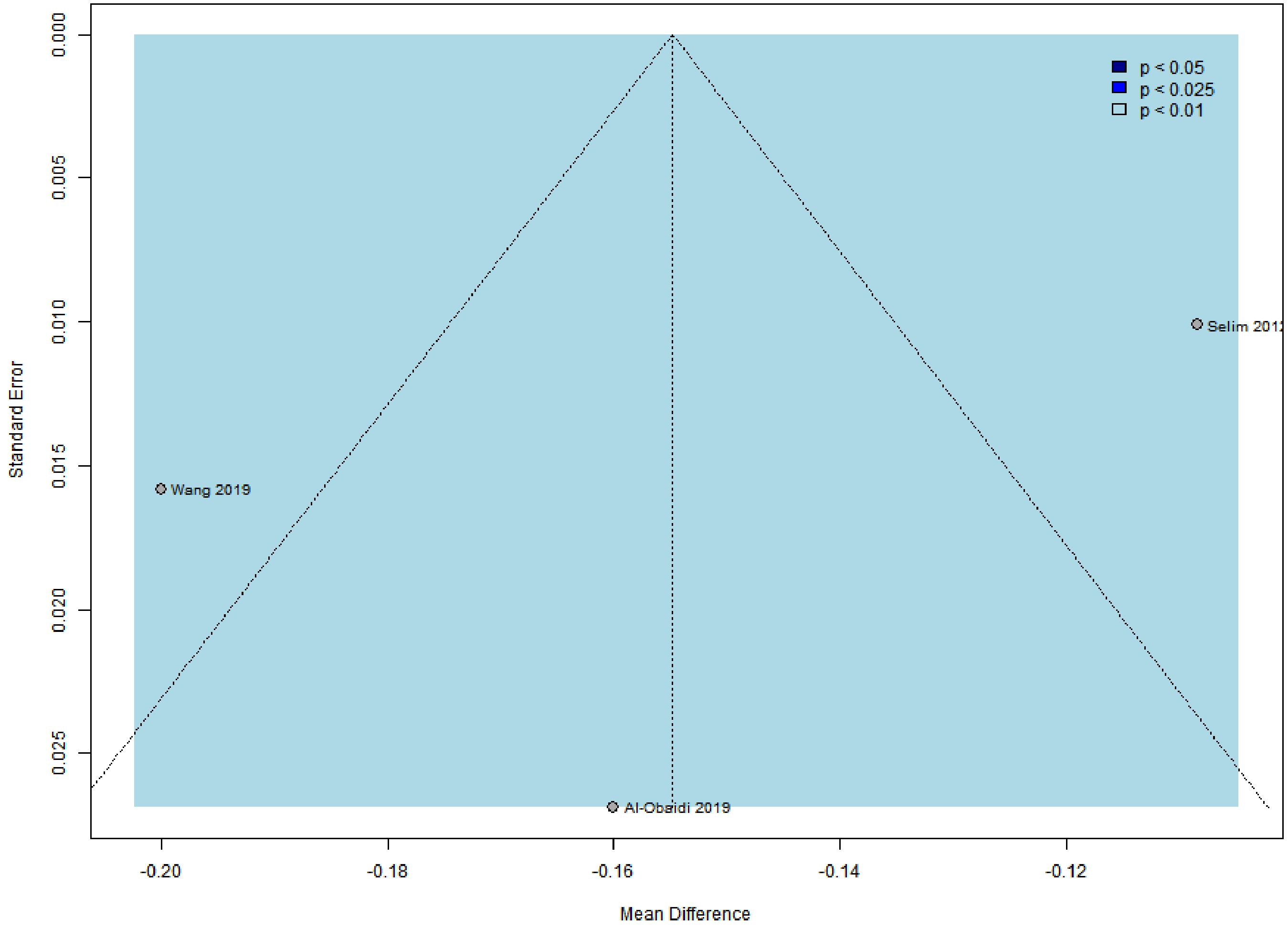

Figure S14. Funnel plot for pulsatility index (PI) of subendometrial arteries

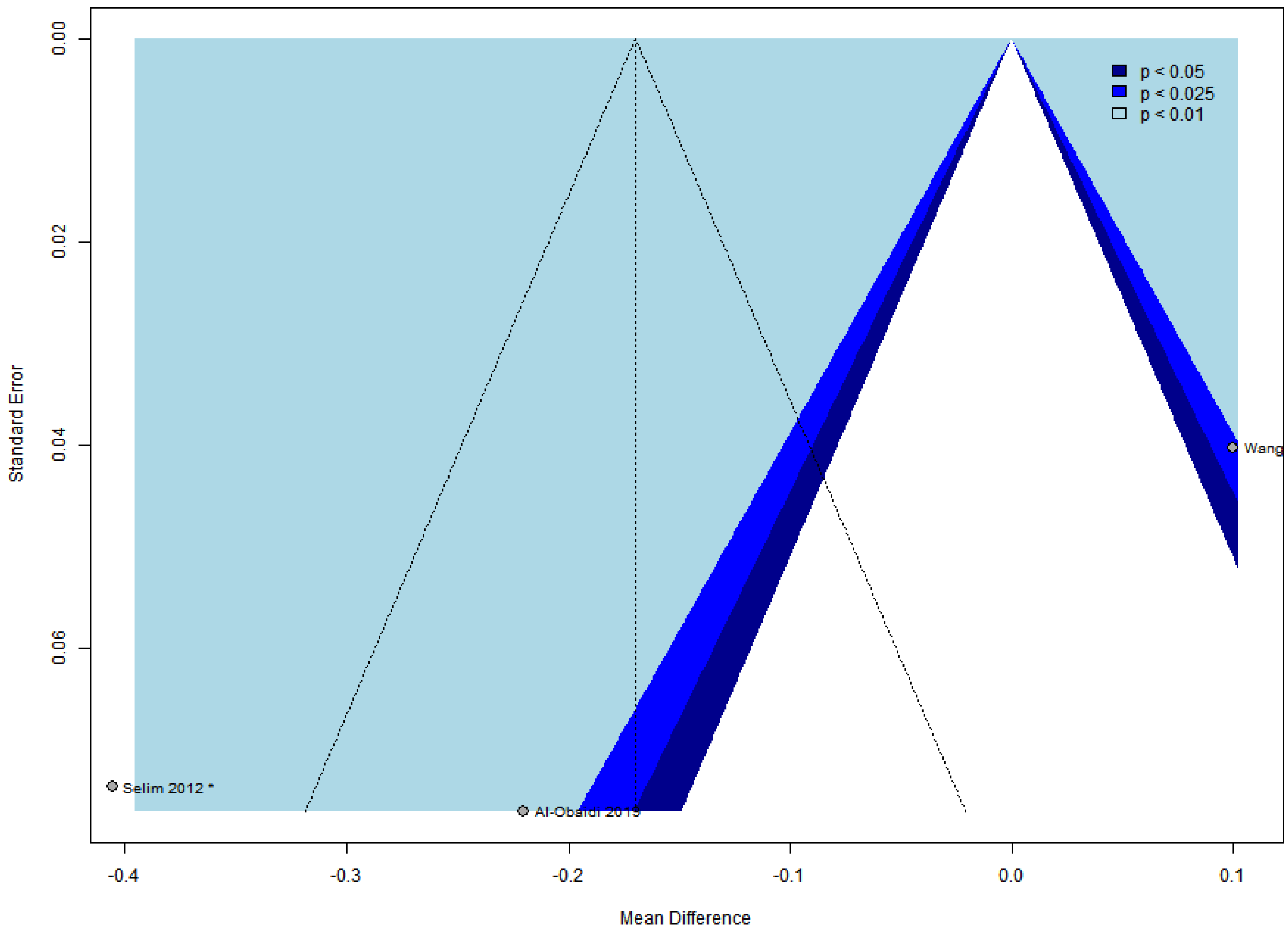

Figure S15. Risk of bias assessment of the studies included in the meta-analysis assessing endometrial thickness (ET) [2-12] using the revised tool for assessing risk of bias in randomized trials (Rob 2)

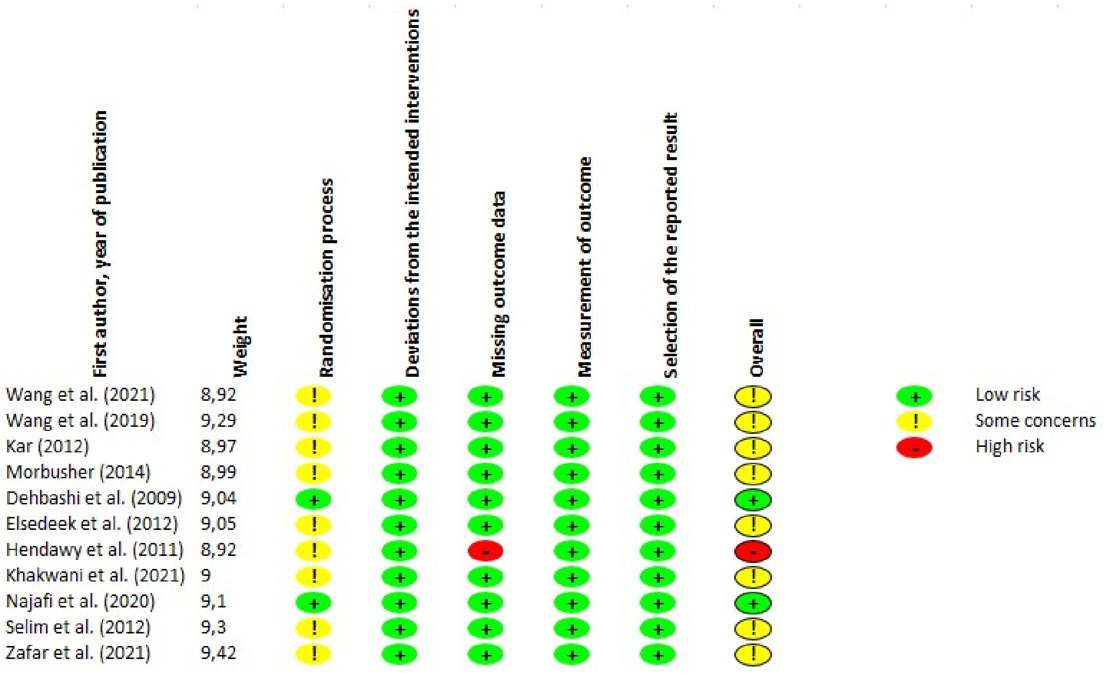

Figure S16. Risk of bias assessment of the studies included in the meta-analysis assessing endometrial thickness (ET) [2-12] broken down to tools, shown in percentage

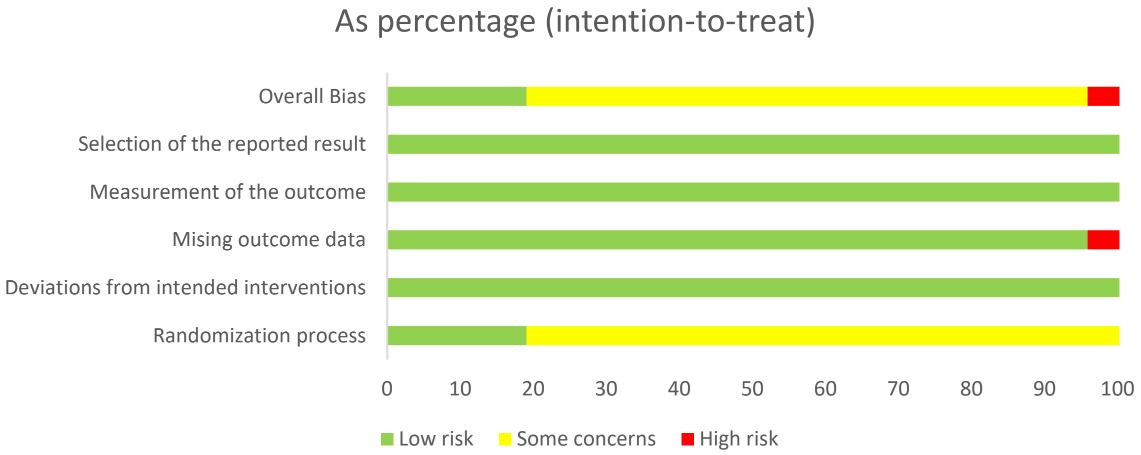

Figure S17. Risk of bias assessment of the studies included in the meta-analysis assessing number of dominant follicles [5-8,10-14] using the revised tool for assessing risk of bias in randomized trials (Rob 2)

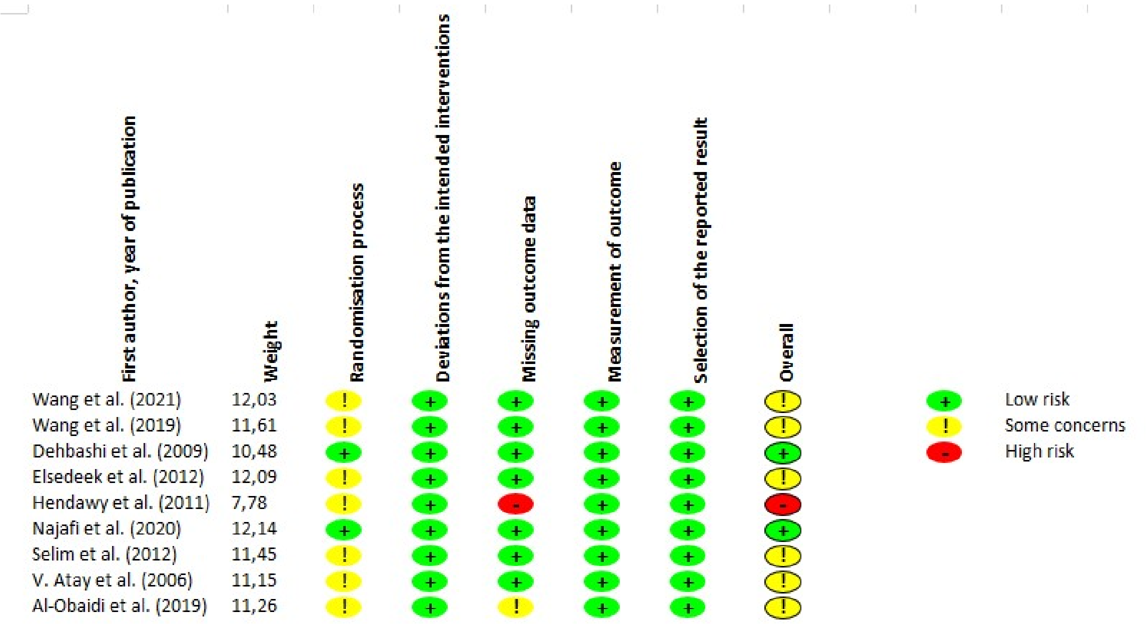

Figure S18. Risk of bias assessment of the studies included in the meta-analysis assessing number of dominant follicles [5-8,10-14] broken down to tools, shown in percentage

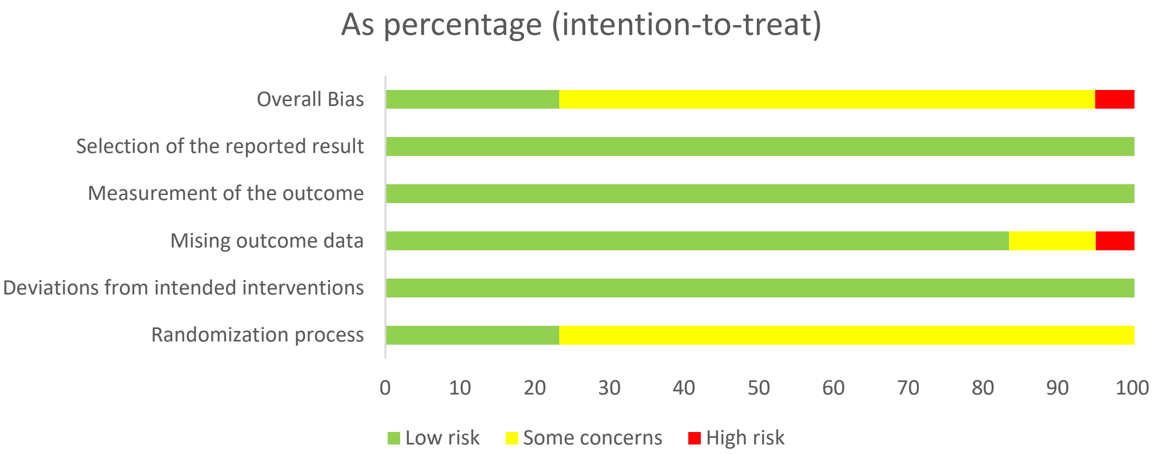

Figure S19. Risk of bias assessment of the studies included in the meta-analysis assessing diameter of dominant follicles [6,7,10,14] using the revised tool for assessing risk of bias in randomized trials (Rob 2)

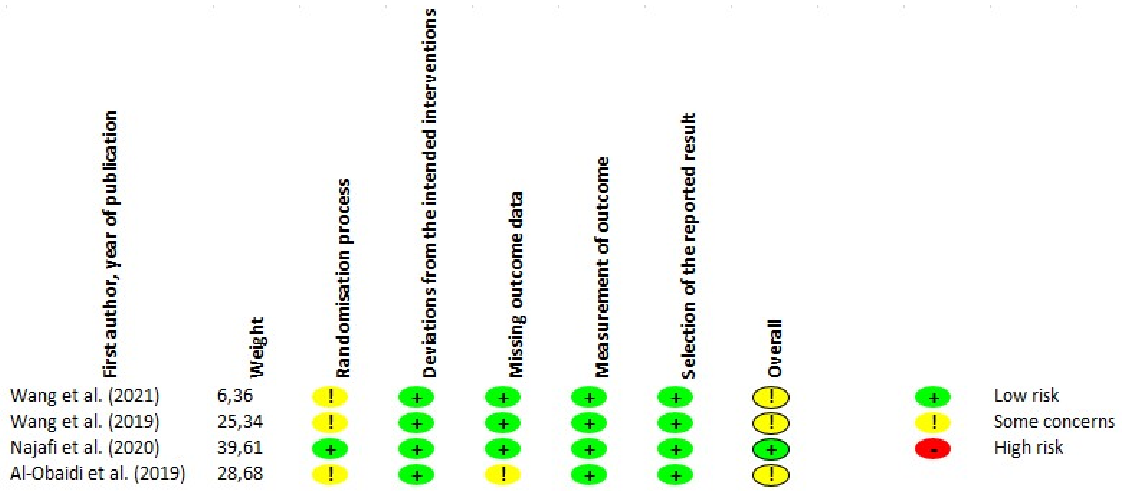

Figure S20. Risk of bias assessment of the studies included in the meta-analysis assessing diameter of dominant follicles [6,7,10,14] broken down to tools, shown in percentage

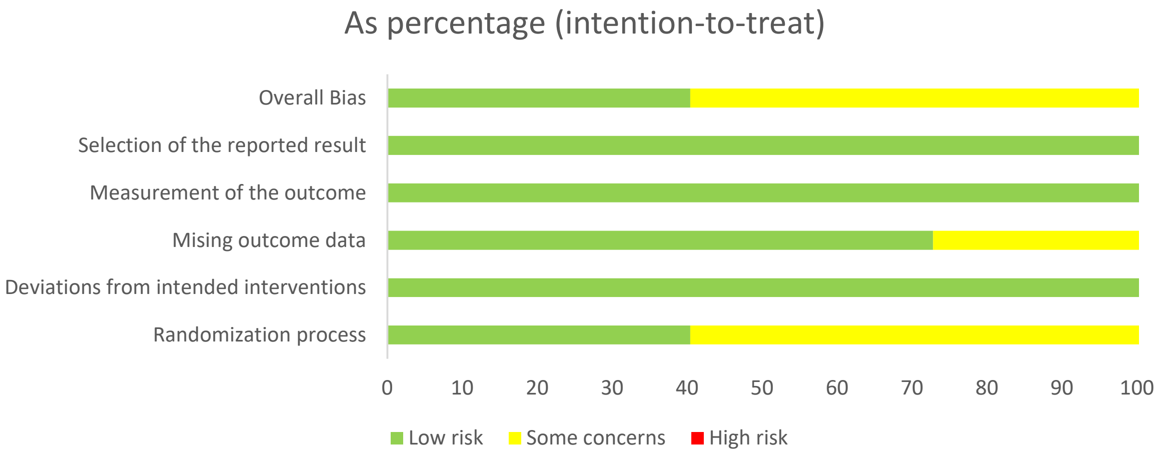

Figure S21. Risk of bias assessment of the studies included in the meta-analysis assessing mono-and multifollicular development [2,3,9] rate using the revised tool for assessing risk of bias in randomized trials (Rob 2)

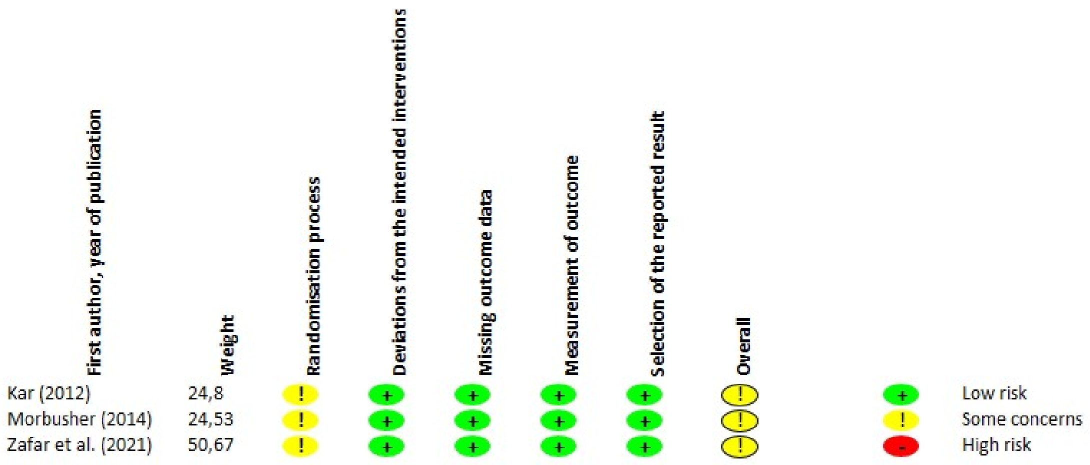

Figure S22. Risk of bias assessment of the studies included in the meta-analysis assessing mono-and multifollicular development rate [2,3,9] broken down to tools, shown in percentage

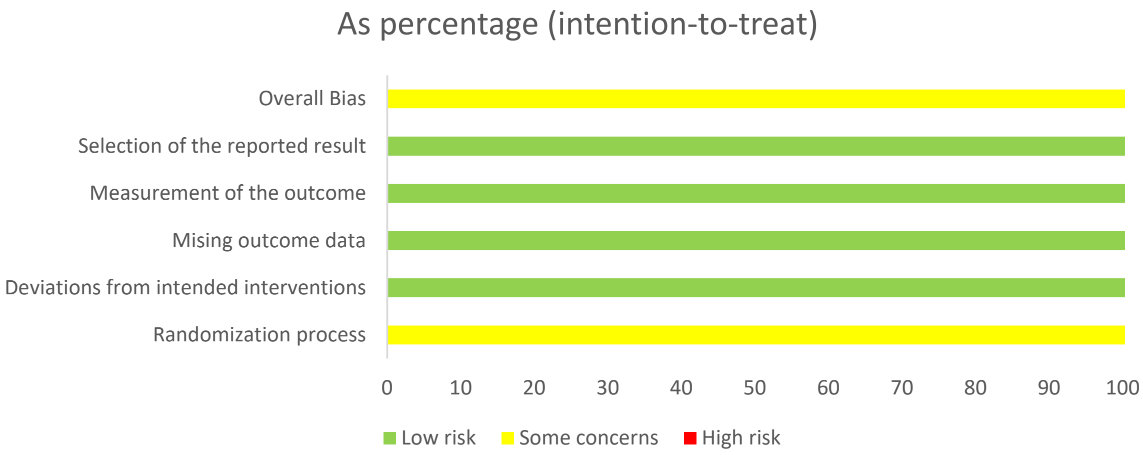

Figure S23. Risk of bias assessment of the studies included in the meta-analysis assessing ovulation rate [2-6,10-12,15] using the revised tool for assessing risk of bias in randomized trials (Rob 2)

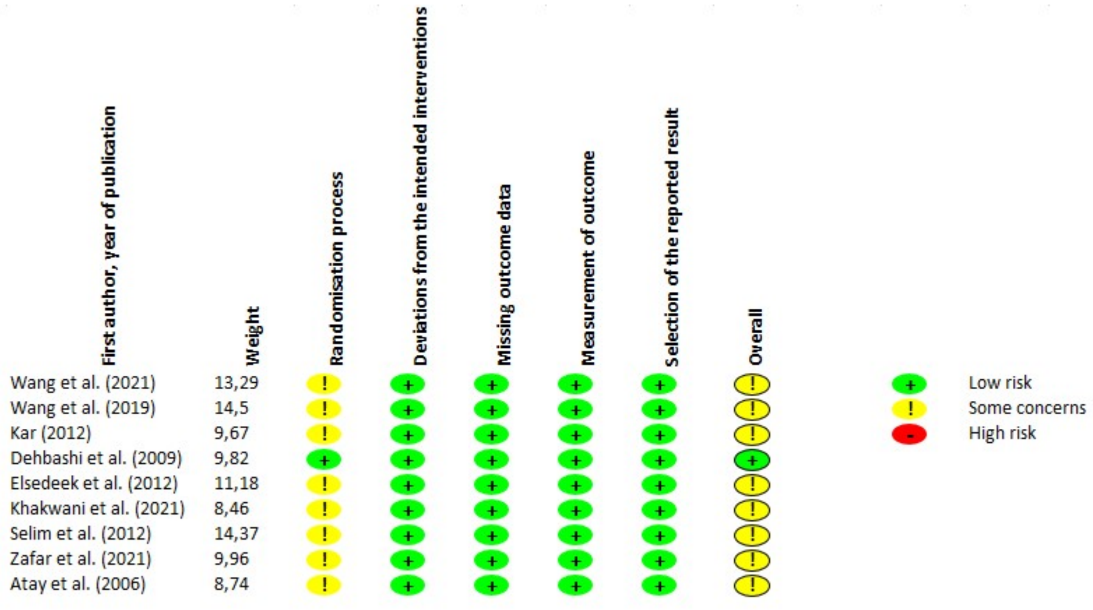

Figure S24. Risk of bias assessment of the studies included in the meta-analysis assessing ovulation rate [2-6,10-12,15] broken down to tools, shown in percentage

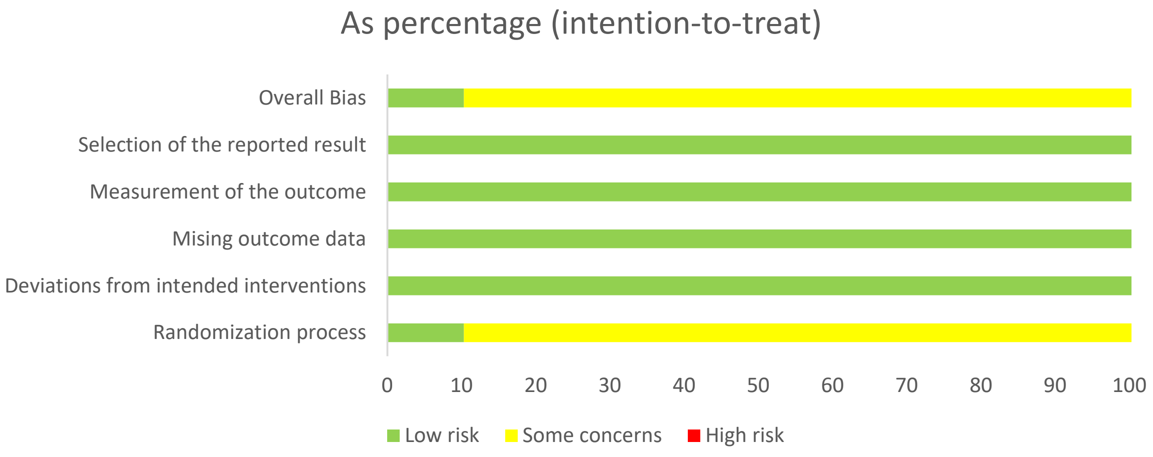

Figure S25. Risk of bias assessment of the studies included in the meta-analysis assessing pregnancy rate [2,5,7,9-16] using the revised tool for assessing risk of bias in randomized trials (Rob 2)

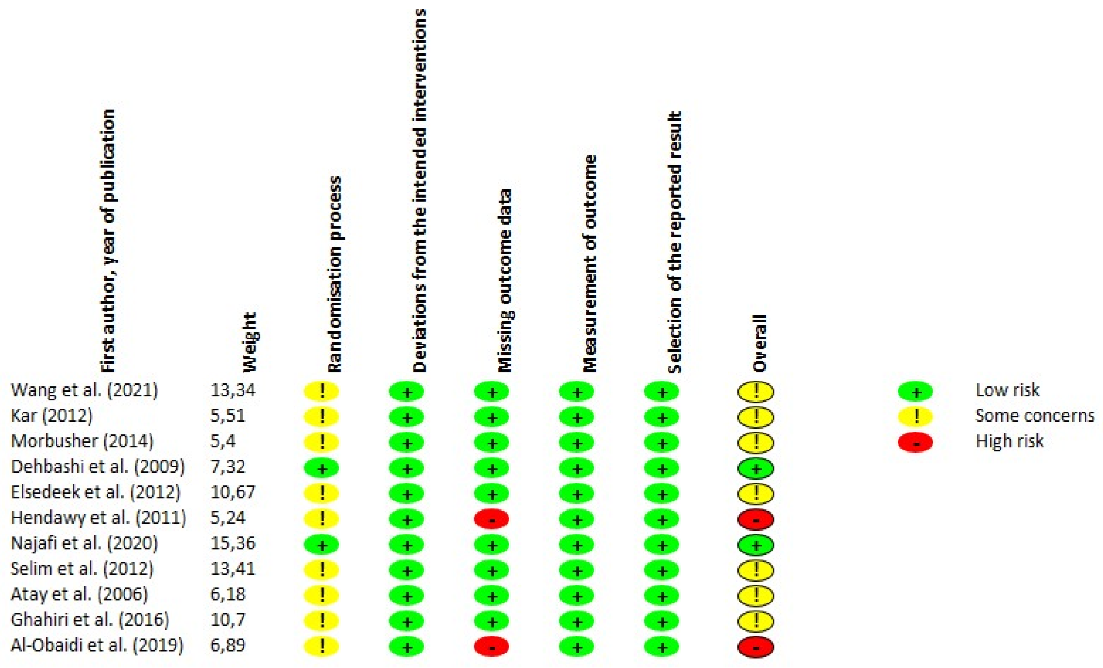

Figure S26. Risk of bias assessment of the studies included in the meta-analysis assessing pregnancy rate [2,5,7,9-16] broken down to tools, shown in percentage

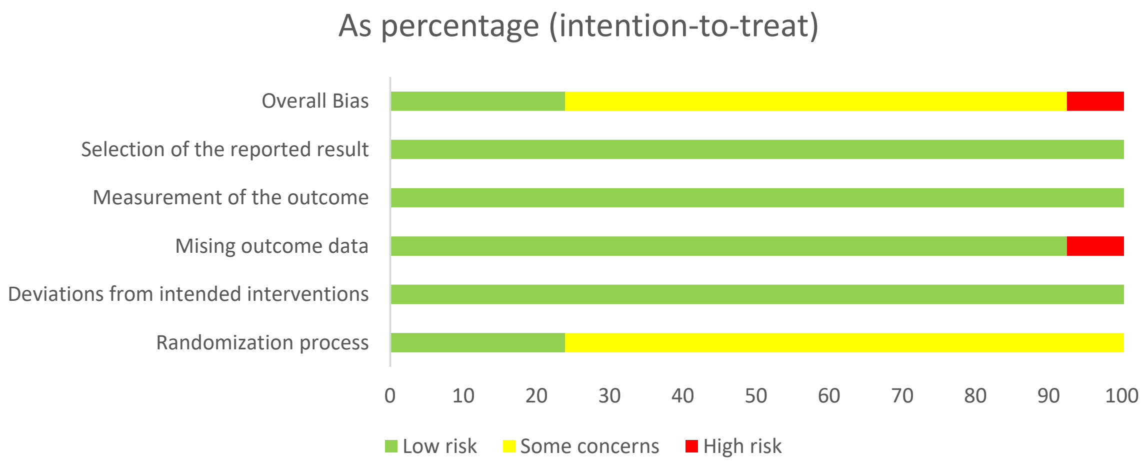

Figure S27. Risk of bias assessment of the studies included in the meta-analysis assessing single and multiple pregnancy rate [5,7-9,11,13,16] using the revised tool for assessing risk of bias in randomized trials (Rob 2)

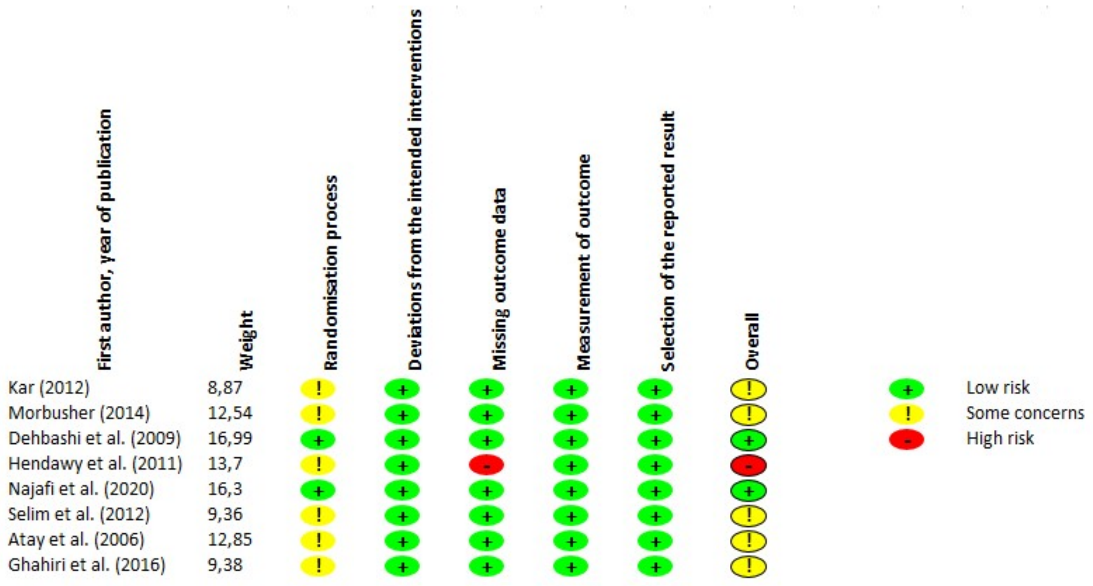

Figure S28. Risk of bias assessment of the studies included in the meta-analysis assessing single and multiple pregnancy rate [5,7-9,11,13,16] broken down to tools, shown in percentage

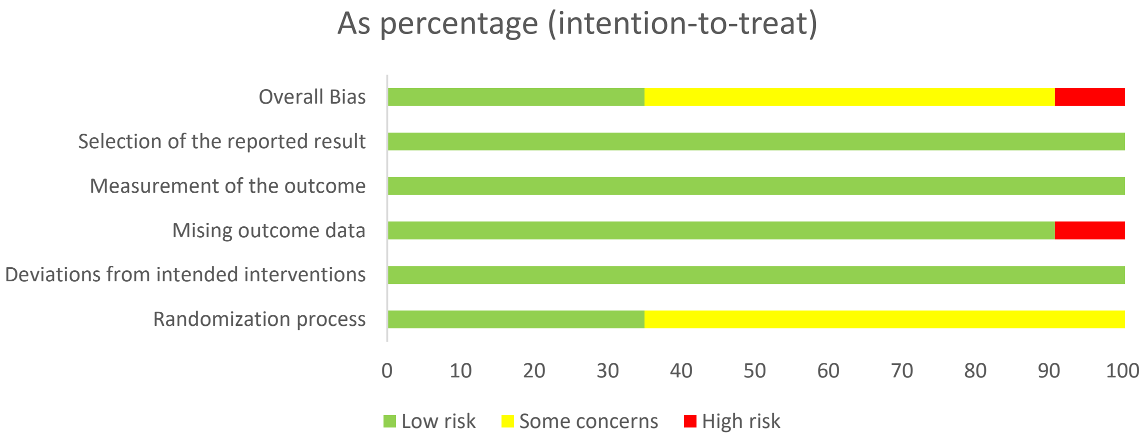

Figure S29. Risk of bias assessment of the studies included in the meta-analysis assessing rate of miscarriage [2,7,11,16] using the revised tool for assessing risk of bias in randomized trials (Rob 2)

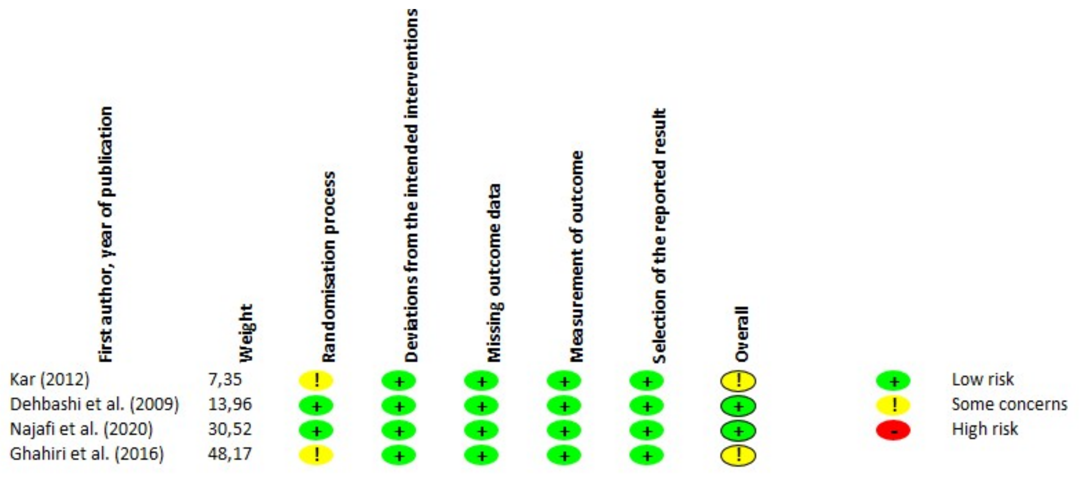

Figure S30. Risk of bias assessment of the studies included in the meta-analysis assessing rate of miscarriage [2,7,11,16] broken down to tools, shown in percentage

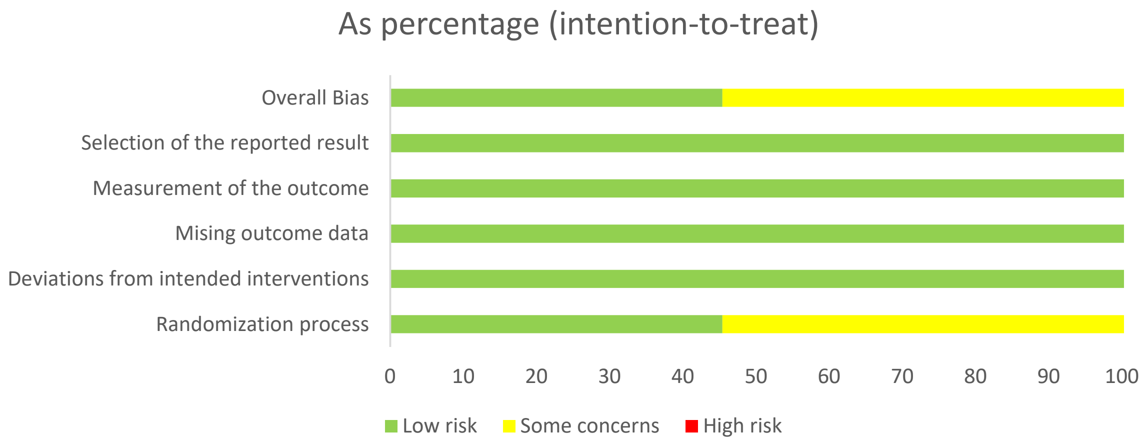

Figure S31. Risk of bias assessment of the studies included in the meta-analysis assessing resistance index (RI) of subendometrial arteries [5,6,14] using the revised tool for assessing risk of bias in randomized trials (Rob 2)

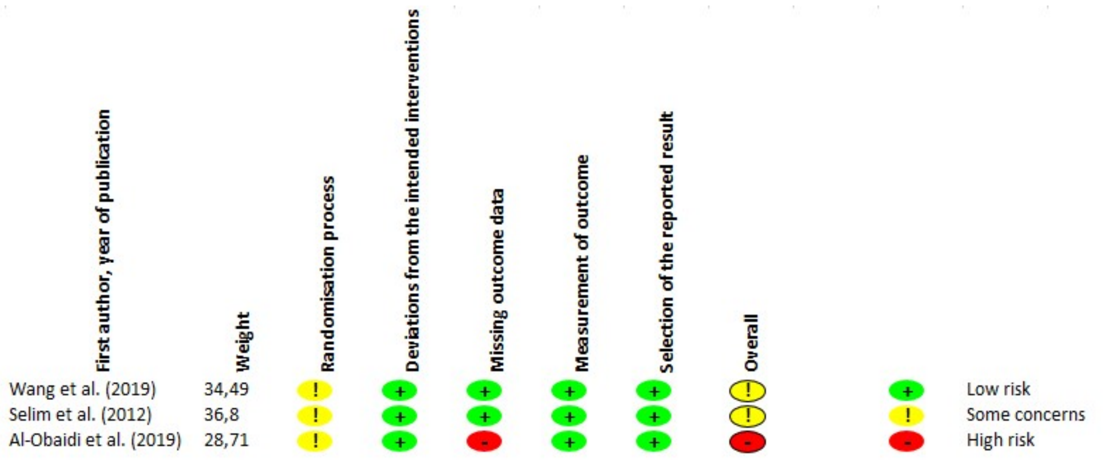

Figure S32. Risk of bias assessment of the studies included in the meta-analysis assessing resistance index (RI) of subendometrial arteries [5,6,14] broken down to tools, shown in percentage

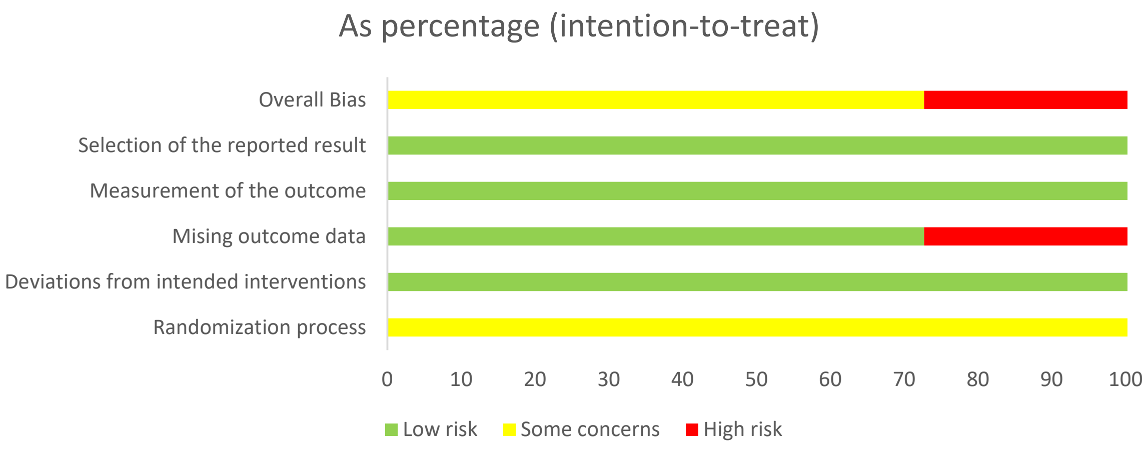

Figure S33. Risk of bias assessment of the studies included in the meta-analysis assessing pulsatility index (PI) of subendometrial arteries [5,6,14] using the revised tool for assessing risk of bias in randomized trials (Rob 2)

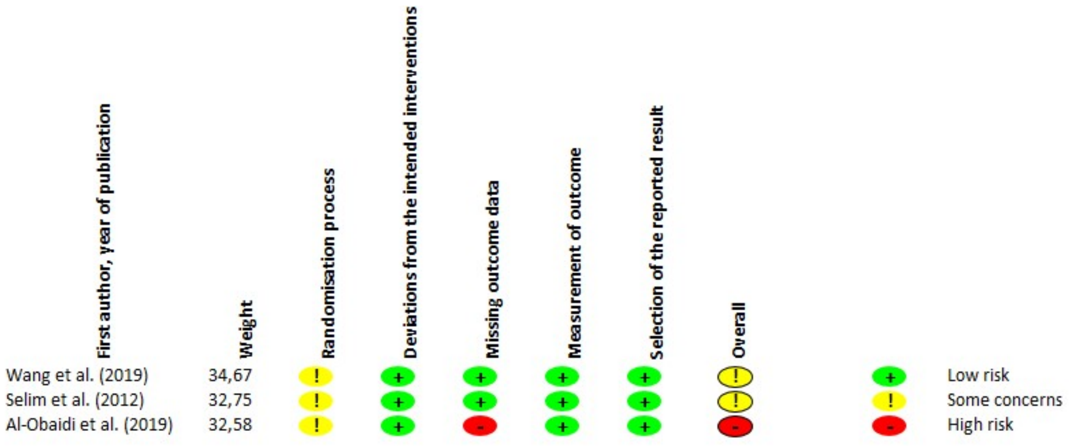

Figure S34. Risk of bias assessment of the studies included in the meta-analysis assessing pulsatility index (PI) of subendometrial arteries of subendometrial arteries [5,6,14] broken down to tools, shown in percentage

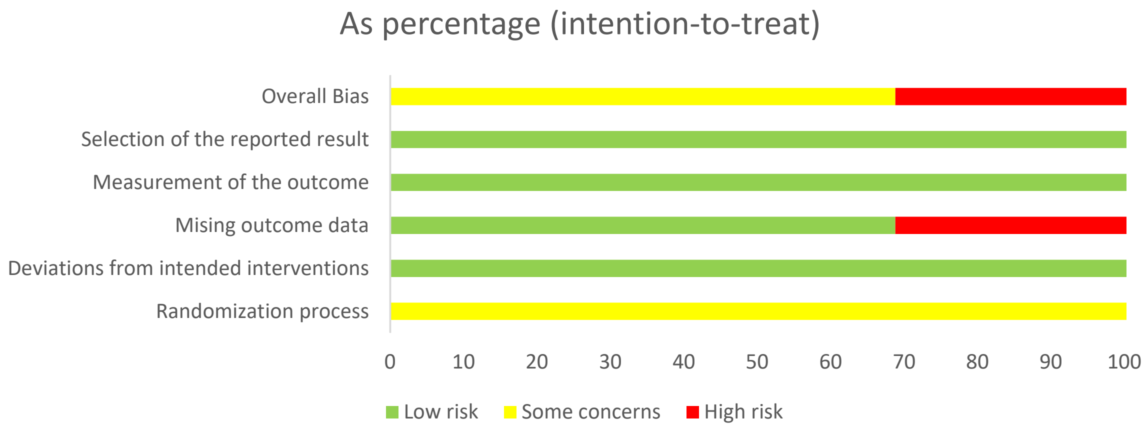

Figure S35. Risk of bias assessment of the studies included in the systematic review assessing rate of endometrial thickness (ET) [14,15,17-25] using the revised tool for assessing risk of bias in randomized trials (Rob 2)

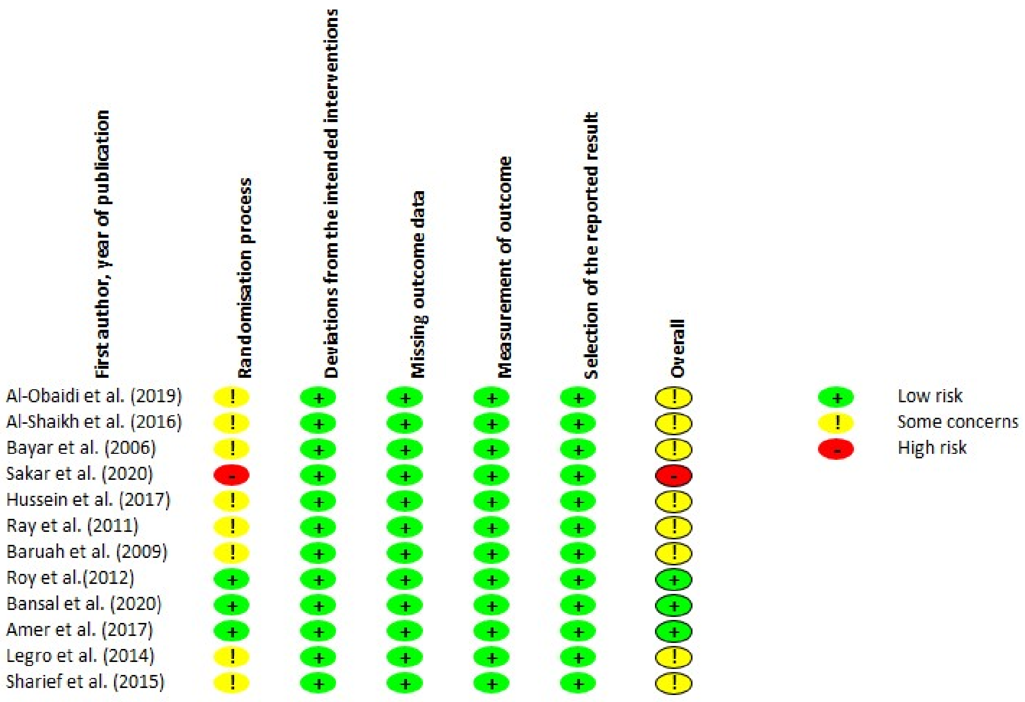

Figure S36. Risk of bias assessment of the studies included in the systematic review assessing rate of endometrial thickness [14,15,17-25] broken down to tools, shown in percentage

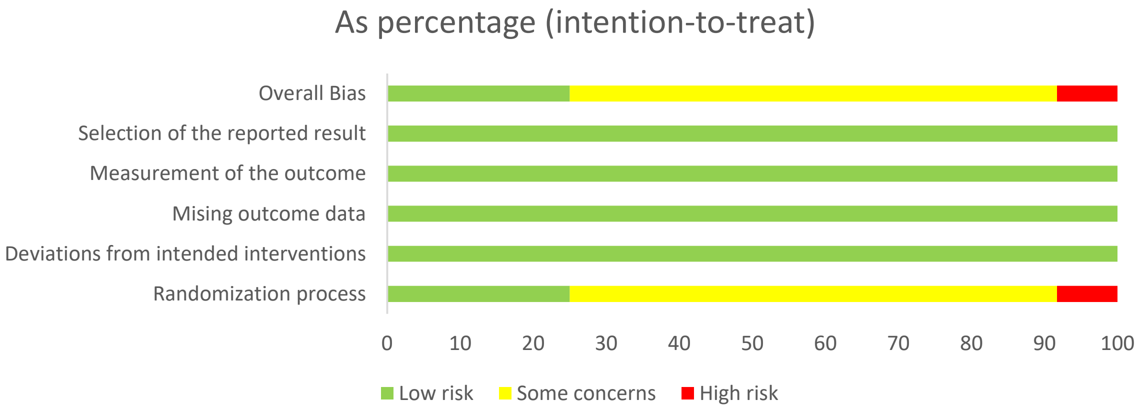

Figure S37. Risk of bias assessment of the studies included in the systematic review assessing endometrial volume (EV) [6,10] using the revised tool for assessing risk of bias in randomized trials (Rob 2)

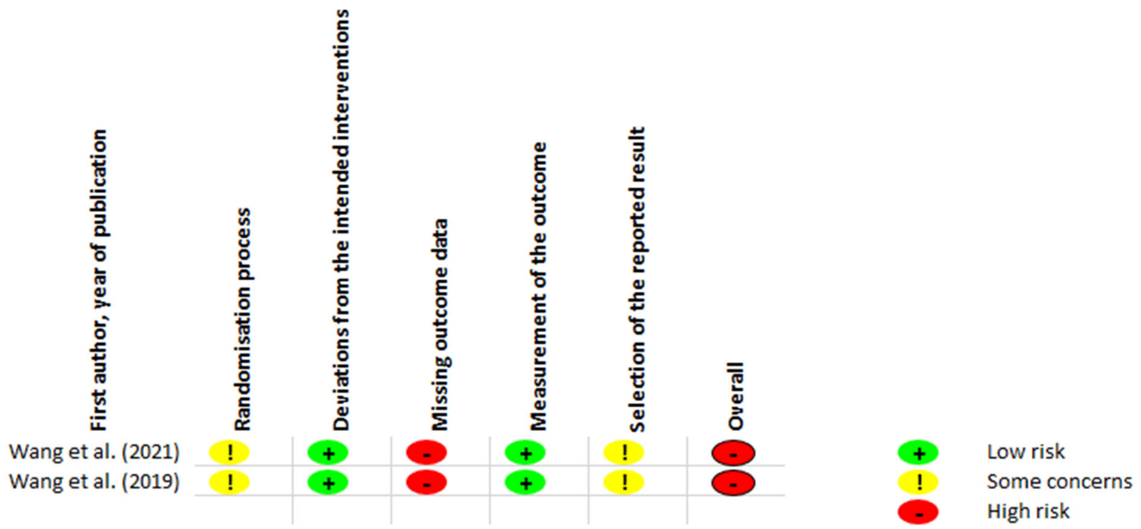

Figure S38. Risk of bias assessment of the studies included in the systematic review assessing endometrial volume (EV) [6,10] broken down to tools, shown in percentage

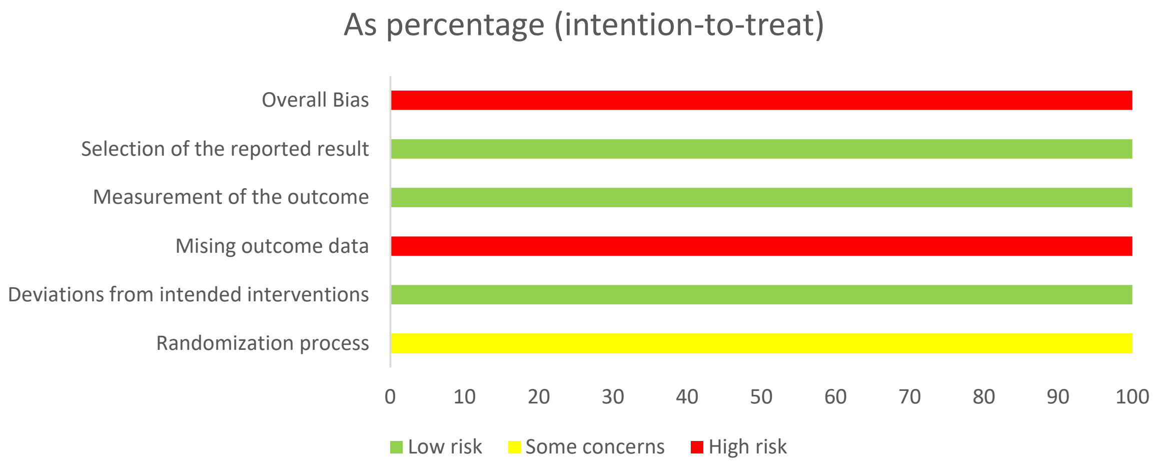

Figure S39. Risk of bias assessment of the studies included in the systematic review assessing endometrial pattern and/or echogenicity [6,15,17] using the revised tool for assessing risk of bias in randomized trials (Rob 2)

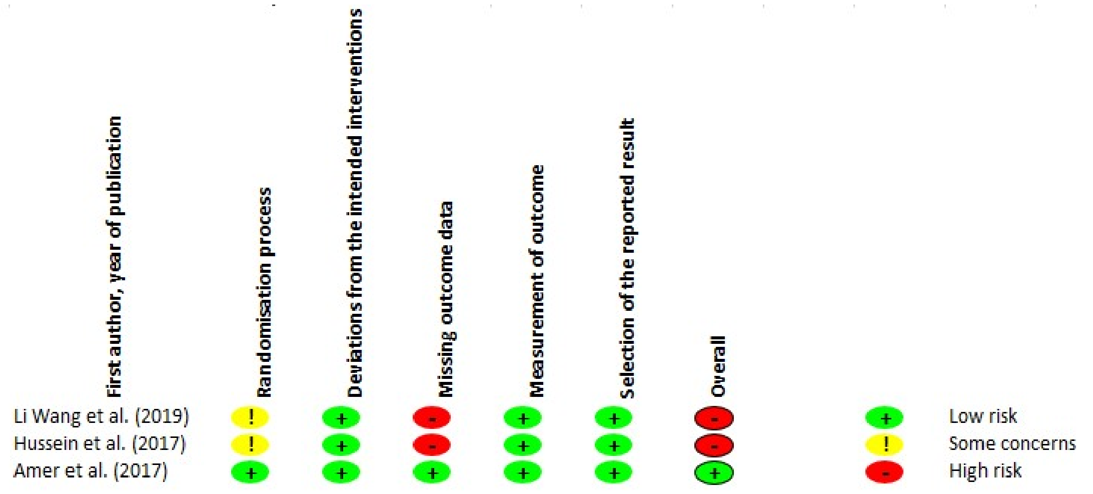

Figure S40. Risk of bias assessment of the studies included in the systematic review assessing endometrial pattern and/or echogenicity [6,15,17] broken down to tools, shown in percentage

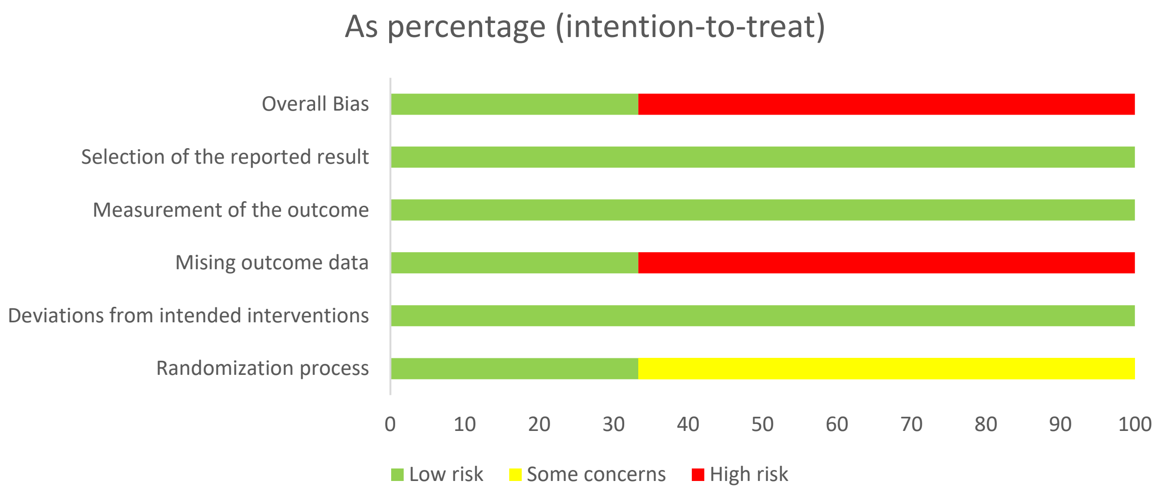

Figure S41. Risk of bias assessment of the studies included in the systematic review assessing rate of number of dominant follicles [18-24] using the revised tool for assessing risk of bias in randomized trials (Rob 2)

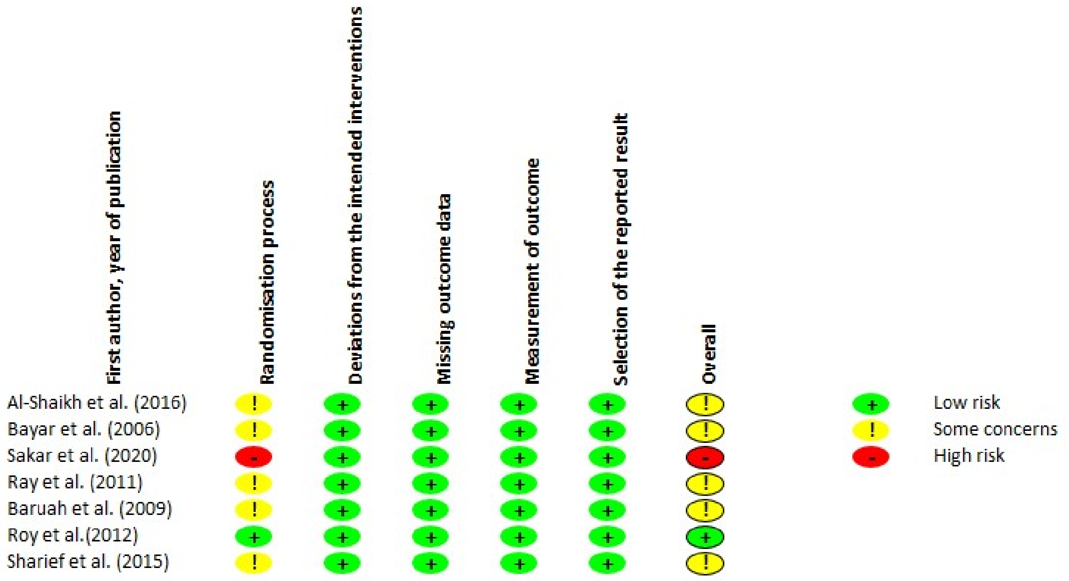

Figure S42. Risk of bias assessment of the studies included in the systematic review assessing rate of number of dominant follicles [18-24] broken down to tools, shown in percentage

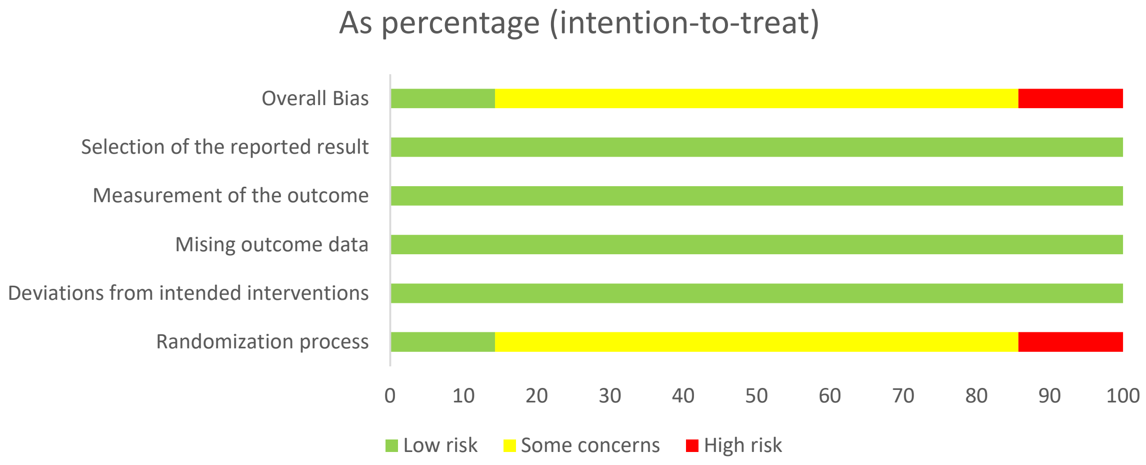

Figure S43. Risk of bias assessment of the studies included in the systematic review assessing rate of diameter of dominant follicles [23,24] using the revised tool for assessing risk of bias in randomized trials (Rob 2)

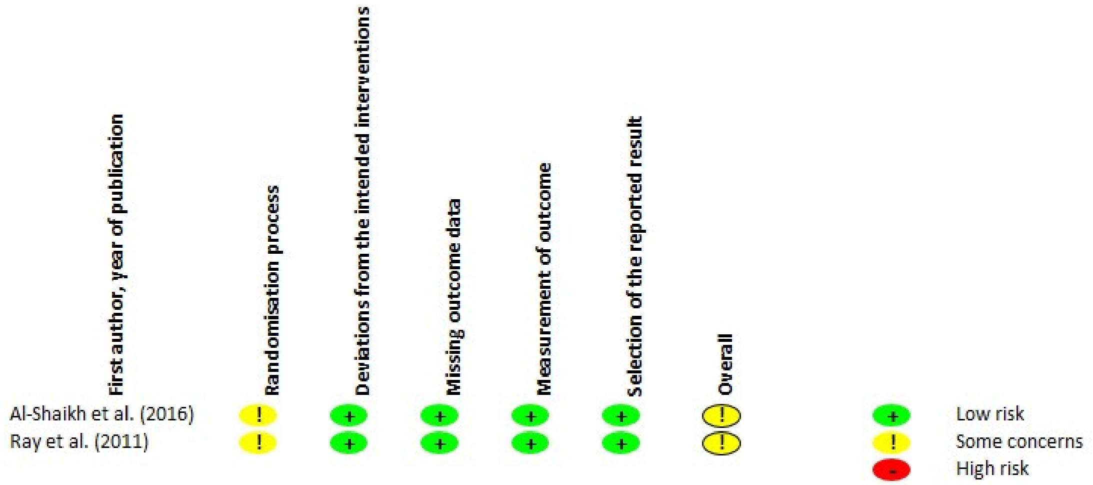

Figure S44. Risk of bias assessment of the studies included in the systematic review assessing rate of diameter of dominant follicles [23,24] broken down to tools, shown in percentage

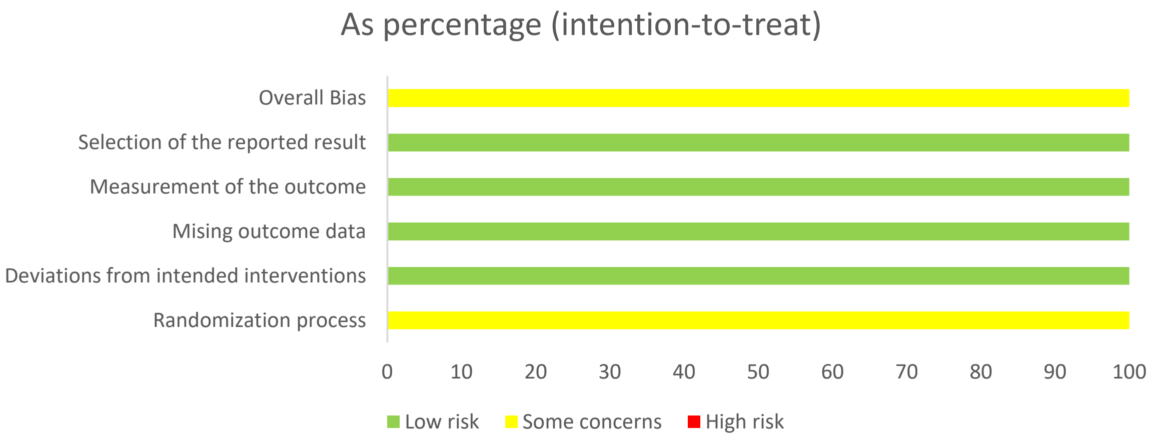

Figure S45. Risk of bias assessment of the studies included in the systematic review assessing monofollicular development cycles [24,25] using the revised tool for assessing risk of bias in randomized trials (Rob 2)

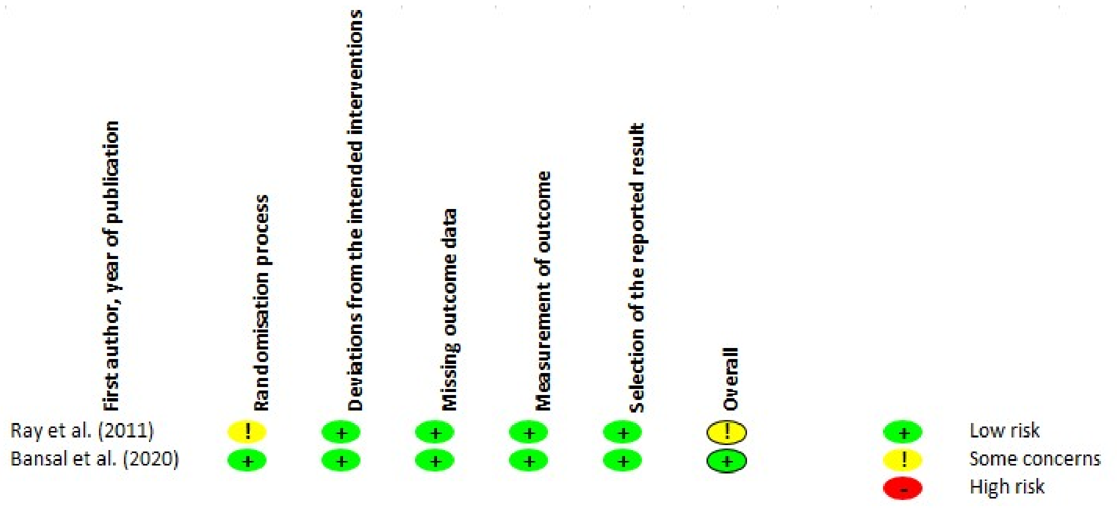

Figure S46. Risk of bias assessment of the studies included in the systematic review assessing monofollicular development cycles [24,25] broken down to tools, shown in percentage

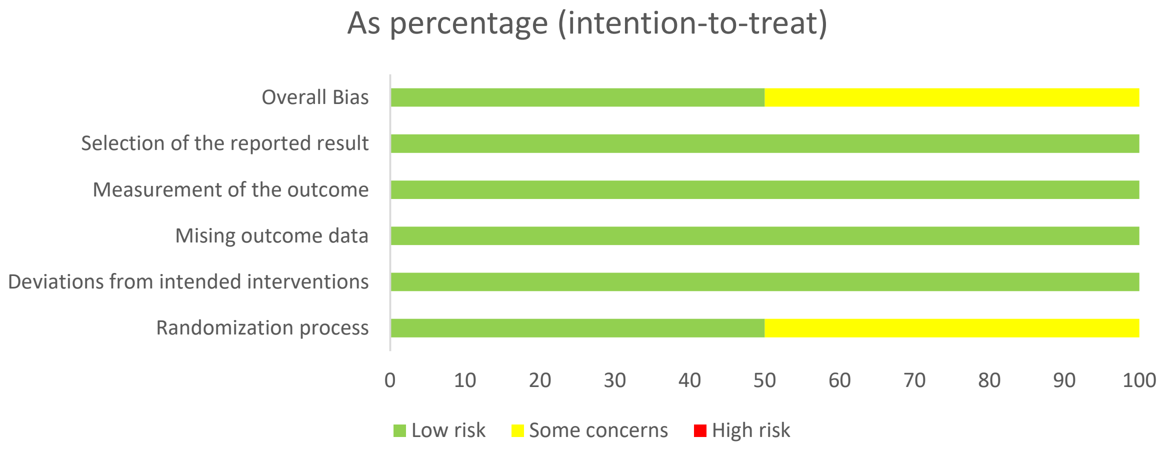

Figure S47. Risk of bias assessment of the studies included in the systematic review assessing ovulation rate [17,20-22,25,26] using the revised tool for assessing risk of bias in randomized trials (Rob 2)

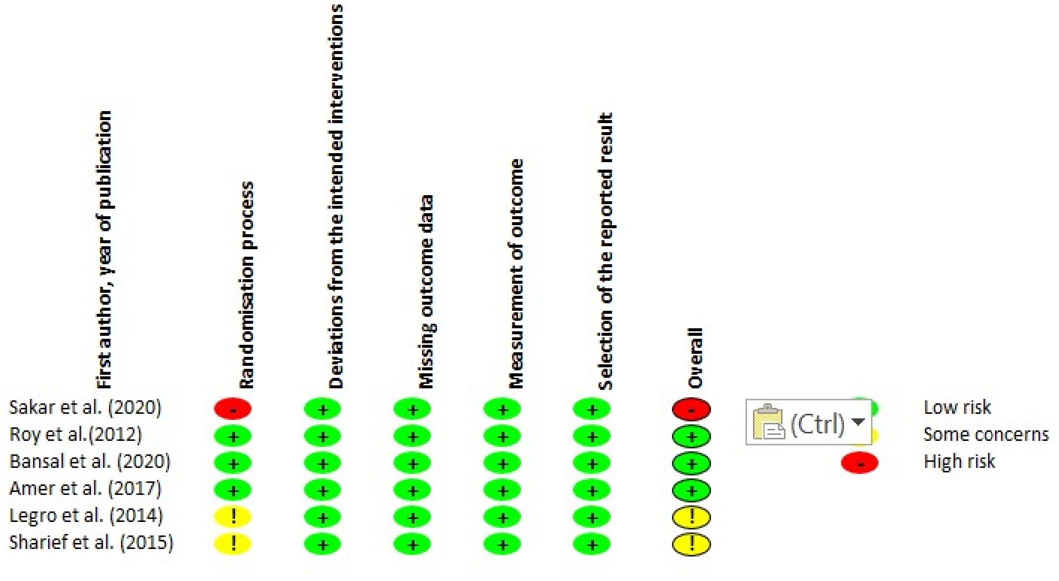

Figure S48. Risk of bias assessment of the studies included in the systematic review assessing ovulation rate [17,20-22,25,26] broken down to tools, shown in percentage

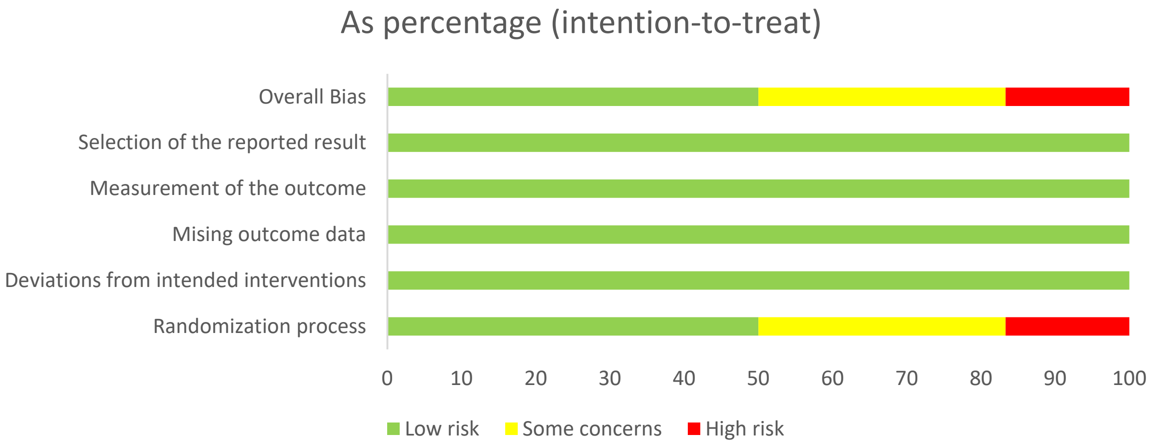

Figure S49. Risk of bias assessment of the studies included in the systematic review assessing pregnancy rate [17-26] using the revised tool for assessing risk of bias in randomized trials (Rob 2)

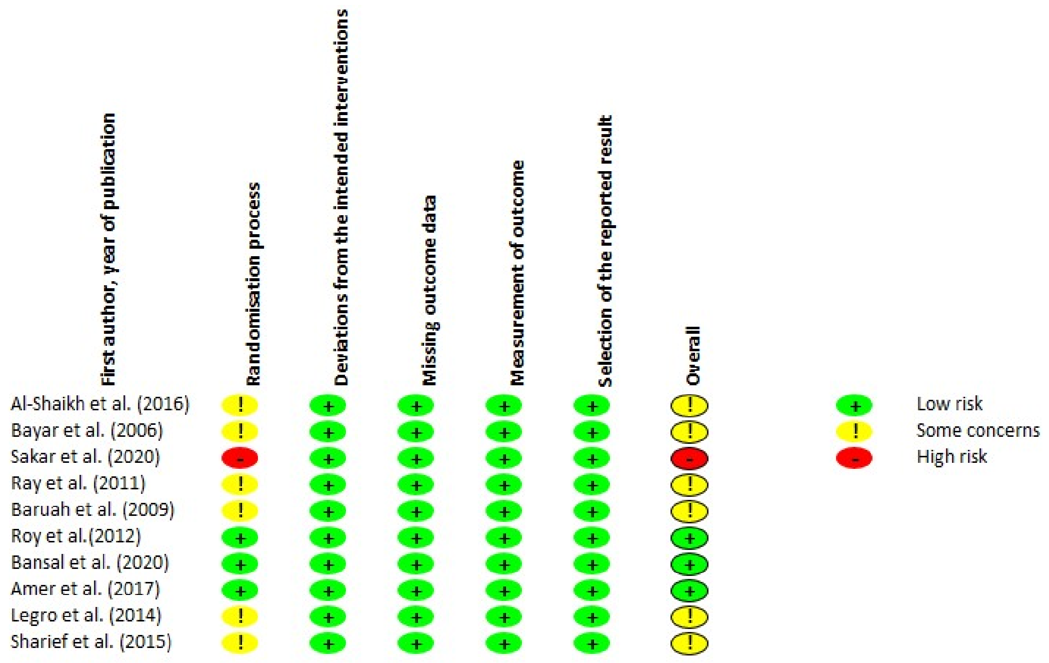

Figure S50. Risk of bias assessment of the studies included in the systematic review assessing pregnancy rate [17-26] broken down to tools, shown in percentage

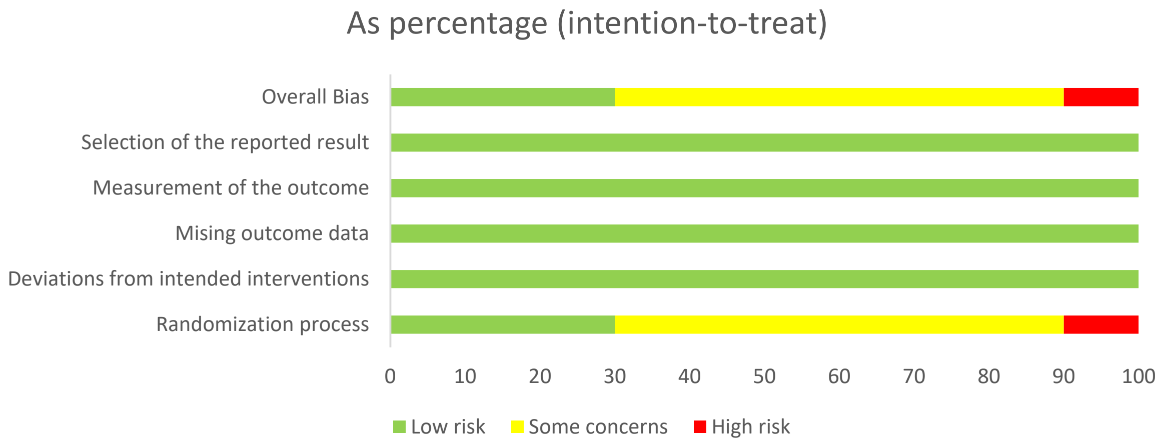

Figure S51. Risk of bias assessment of the studies included in the systematic review assessing number of multiple pregnancies [18-21,26] using the revised tool for assessing risk of bias in randomized trials (Rob 2)

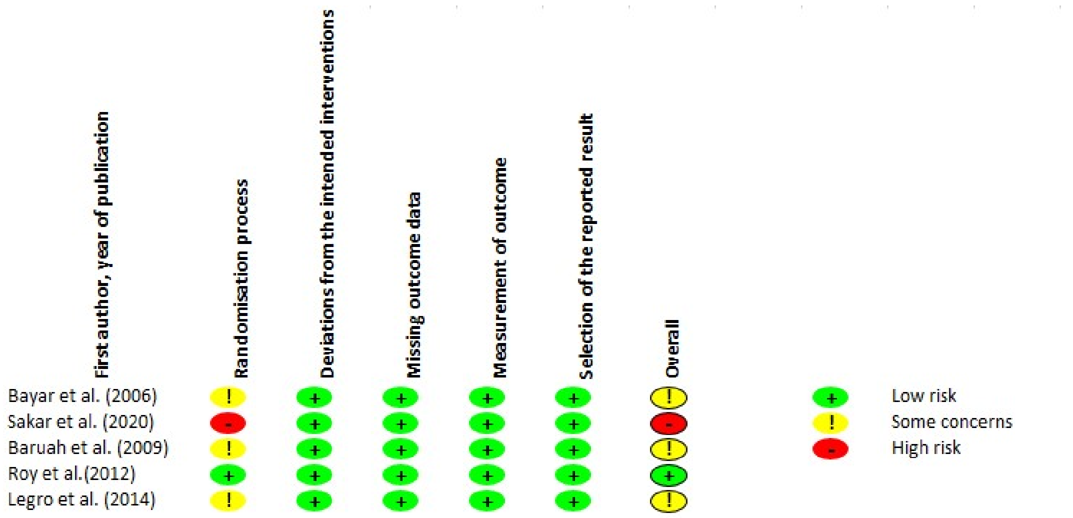

Figure S52. Risk of bias assessment of the studies included in the systematic review assessing number of multiple pregnancies [18-21,26] broken down to tools, shown in percentage

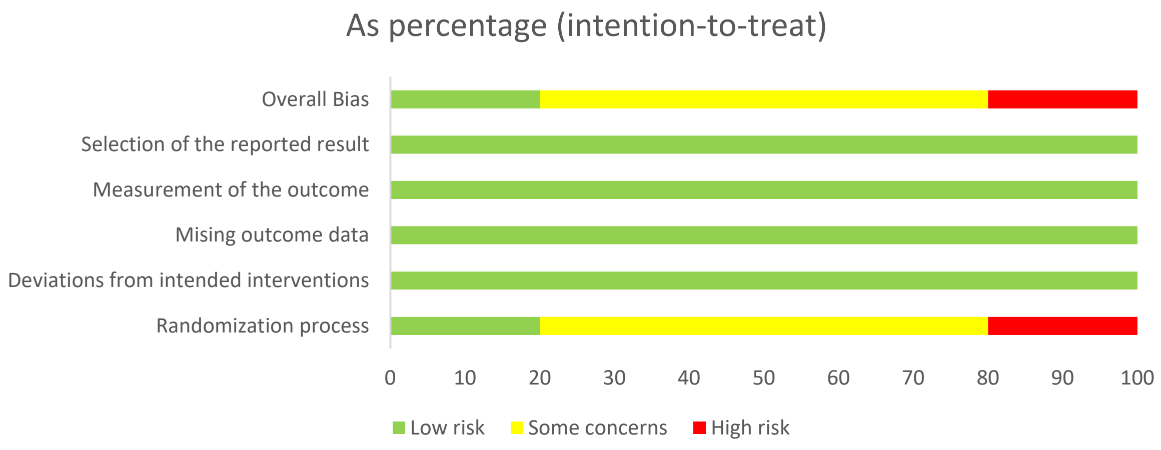

Figure S53. Risk of bias assessment of the studies included in the systematic review assessing number of miscarriages [19-21,23,24,26] using the revised tool for assessing risk of bias in randomized trials (Rob 2)

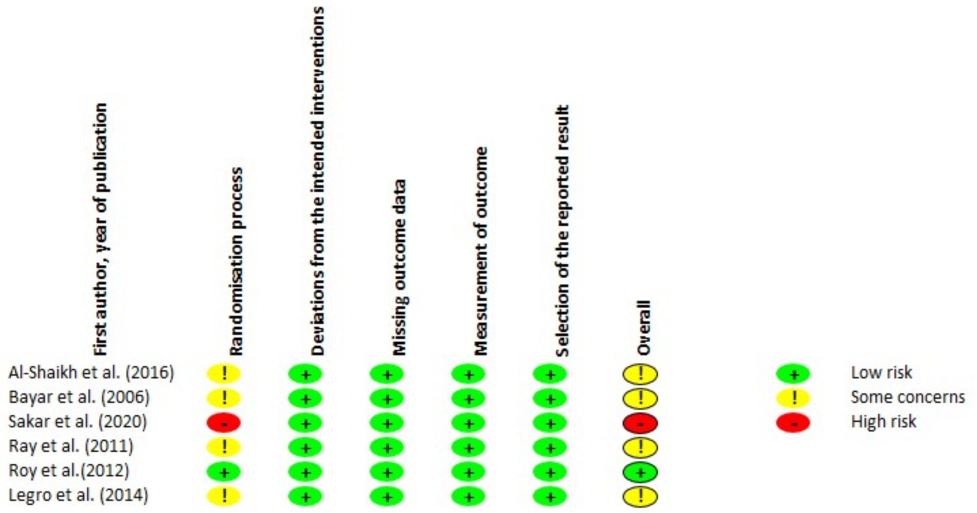

Figure S54. Risk of bias assessment of the studies included in the systematic review assessing number of miscarriages [19-21,23,24,26] broken down to tools, shown in percentage

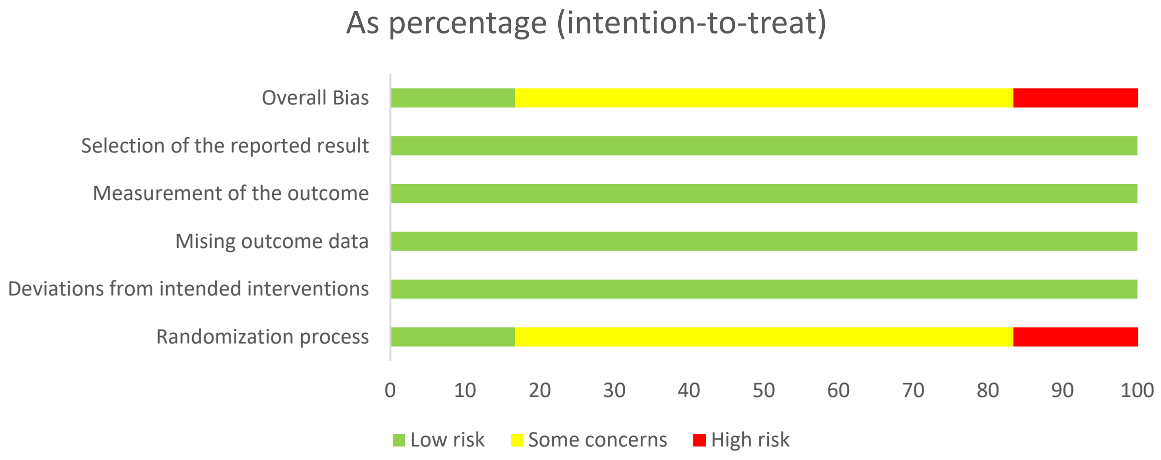

Figure S55. Risk of bias assessment of the studies included in the systematic review assessing live birth rate [11,17,19-21,26] using the revised tool for assessing risk of bias in randomized trials (Rob 2)

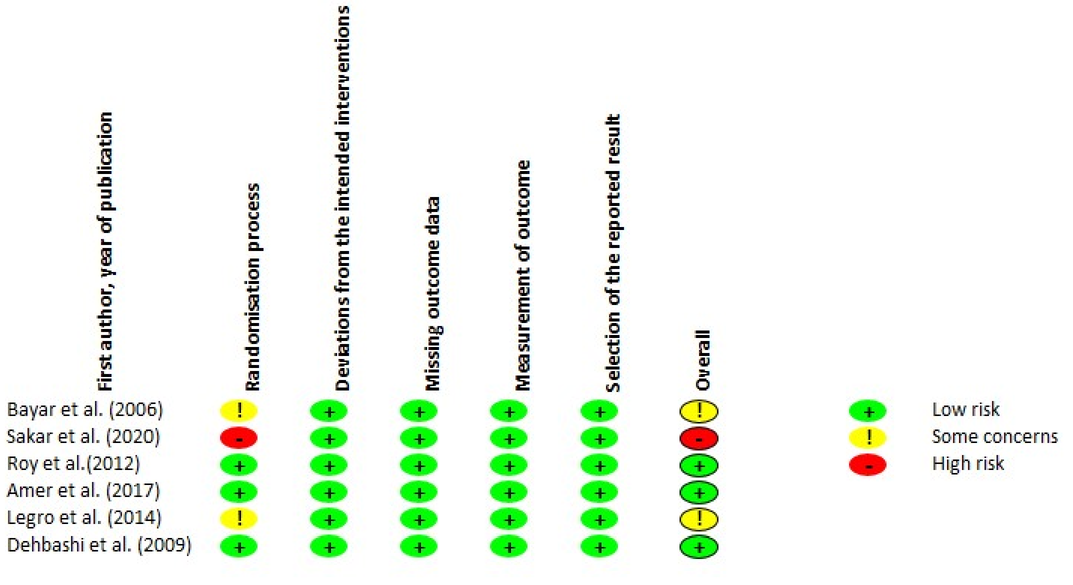

Figure S56. Risk of bias assessment of the studies included in the systematic review assessing live birth rate [11,17,19-21,26] broken down to tools, shown in percentage

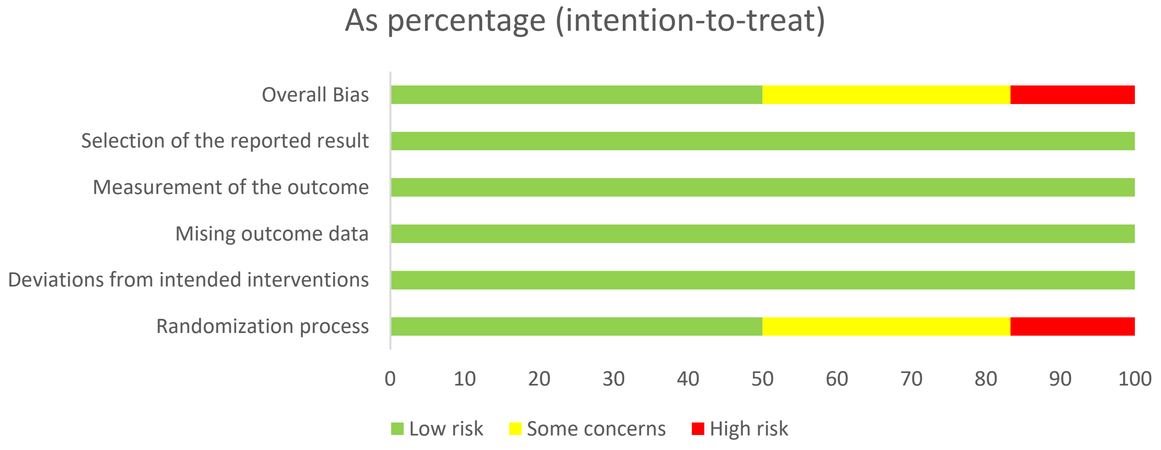

Figure S57. Risk of bias assessment of the studies included in the systematic review assessing number of ectopic pregnancies [16] using the revised tool for assessing risk of bias in randomized trials (Rob 2)

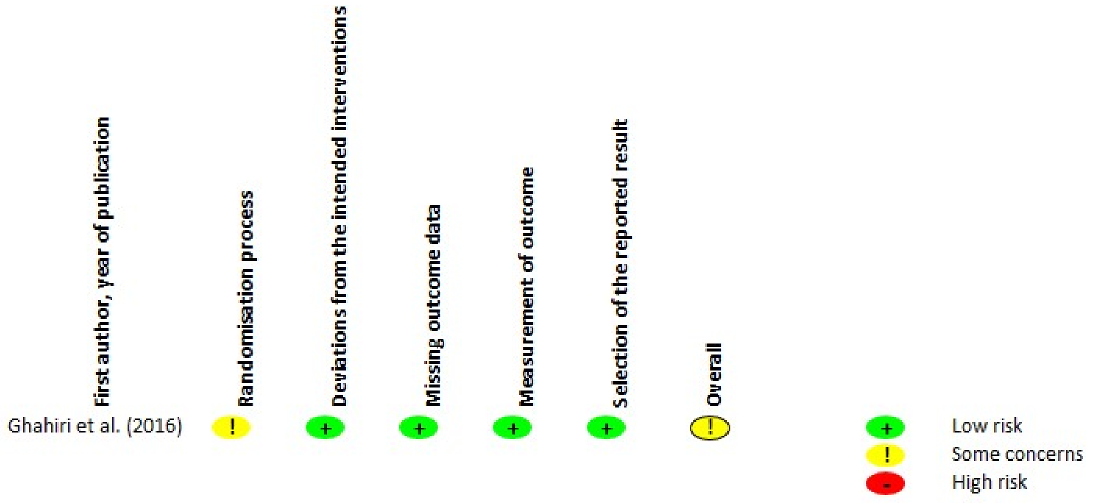

Figure S58. Risk of bias assessment of the studies included in the systematic review assessing number of ectopic pregnancies [16] broken down to tools, shown in percentage

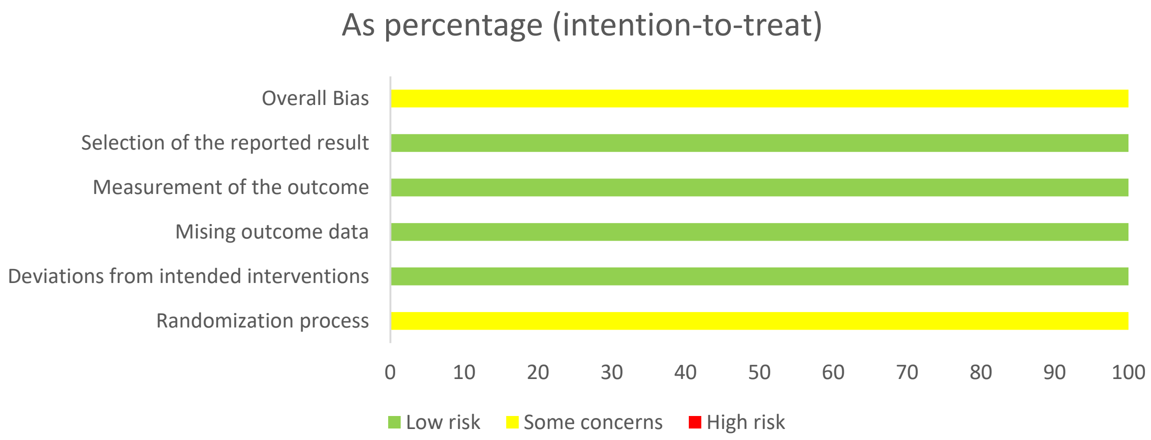

Figure S59. Risk of bias assessment of the studies included in the systematic review assessing number of fetal anomalies [11,17,21,24,26] using the revised tool for assessing risk of bias in randomized trials (Rob 2)

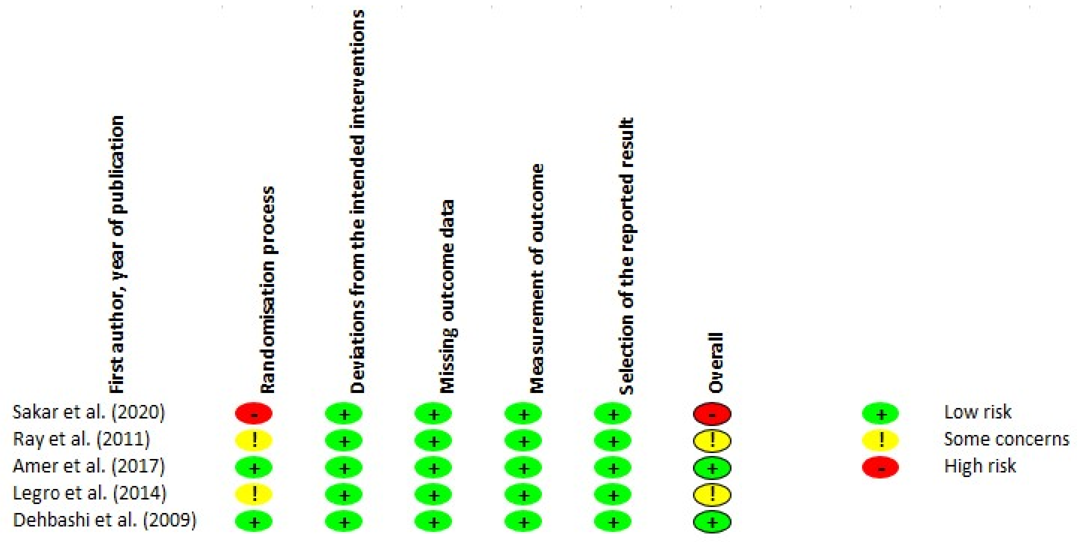

Figure S60. Risk of bias assessment of the studies included in the systematic review assessing number of fetal anomalies [11,17,21,24,26] broken down to tools, shown in percentage

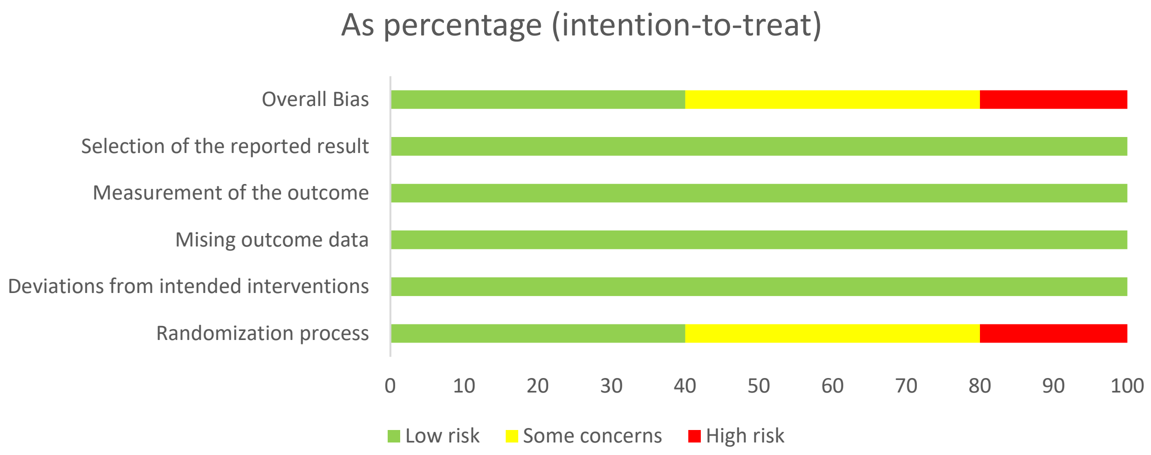

Figure S61. Risk of bias assessment of the studies included in the systematic review assessing endometrial vascularization index (VI), flow index (FI), vascularization flow index (VFI) and detection rate of endometrial-subendometrial blood flow [5,6,10] using the revised tool for assessing risk of bias in randomized trials (Rob 2)

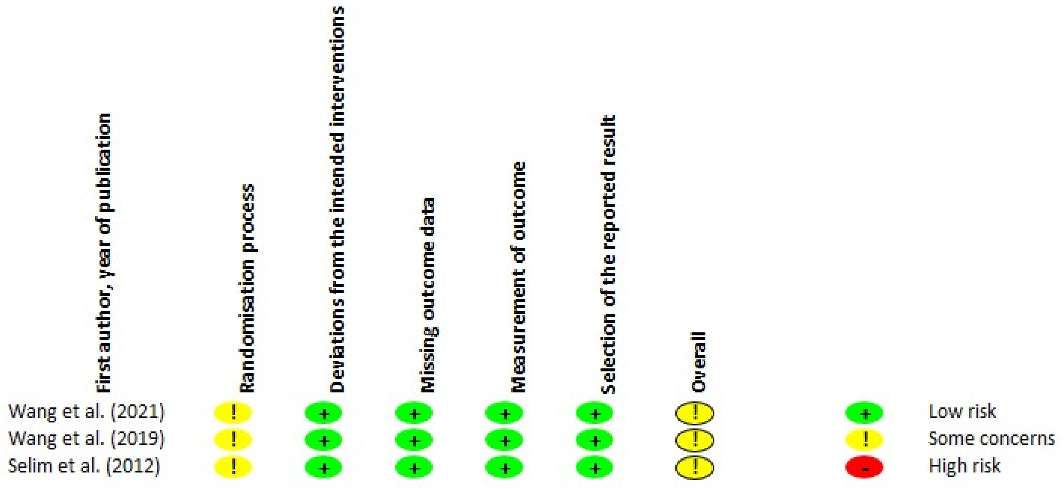

Figure S62. Risk of bias assessment of the studies included in the systematic review assessing endometrial vascularization index (VI), flow index (FI), vascularization flow index (VFI) and detection rate of endometrial-subendometrial blood flow [5,6,10] broken down to tools, shown in percentage

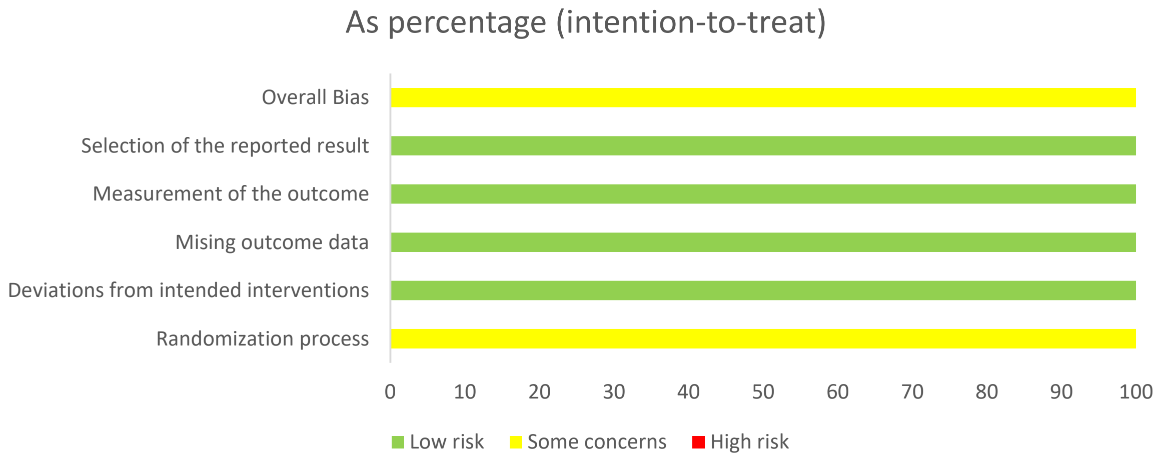

Figure S63. Risk of bias assessment of the studies included in the systematic review assessing systolic velocity (SV)/diastolic velocity (DV) of subendometrial arteries [14] using the revised tool for assessing risk of bias in randomized trials (Rob 2)

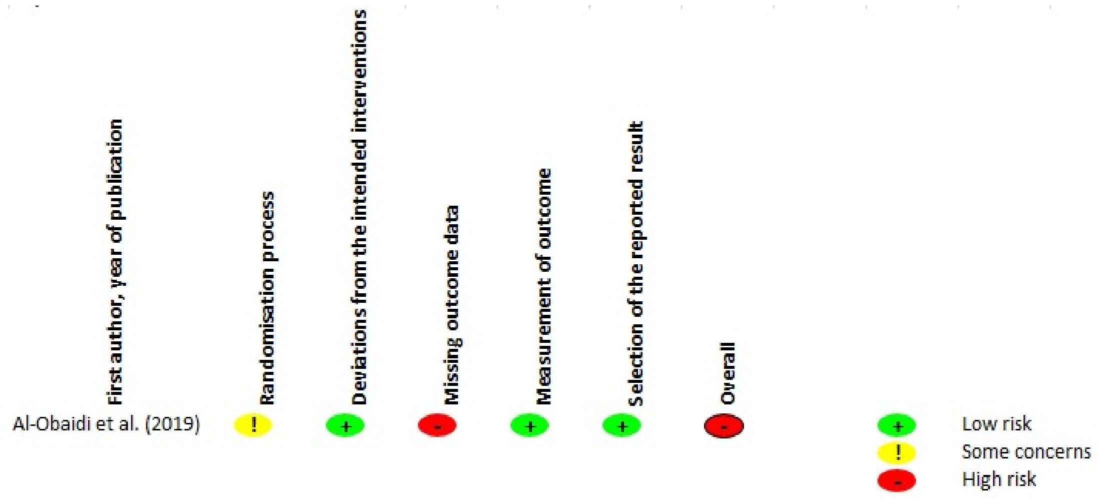

Figure S64. Risk of bias assessment of the studies included in the systematic review assessing systolic velocity (SV)/diastolic velocity (DV) of subendometrial arteries [14] broken down to tools, shown in percentage

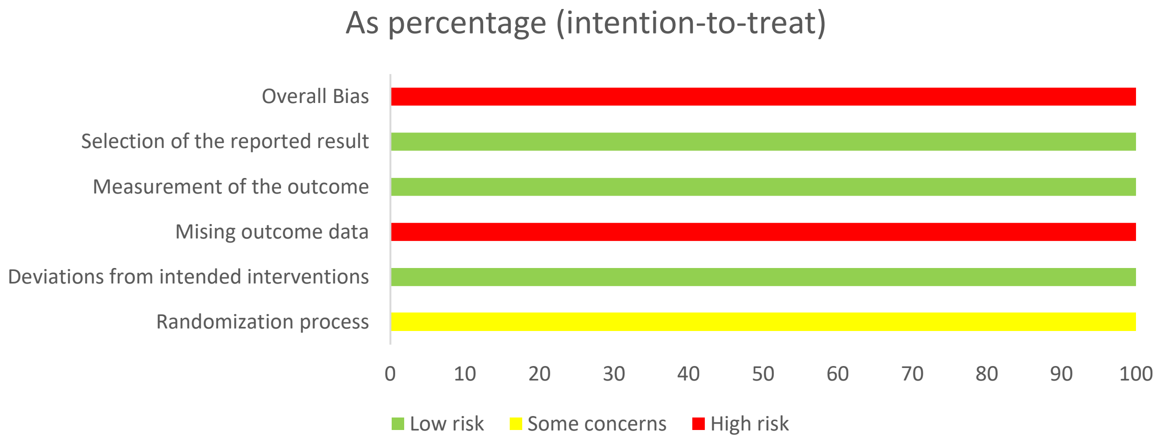

Figure S65. Risk of bias assessment of the studies included in the systematic review assessing resistance index (RI) and pulsatility index (PI) of uterine arteries [6,10] using the revised tool for assessing risk of bias in randomized trials (Rob 2)

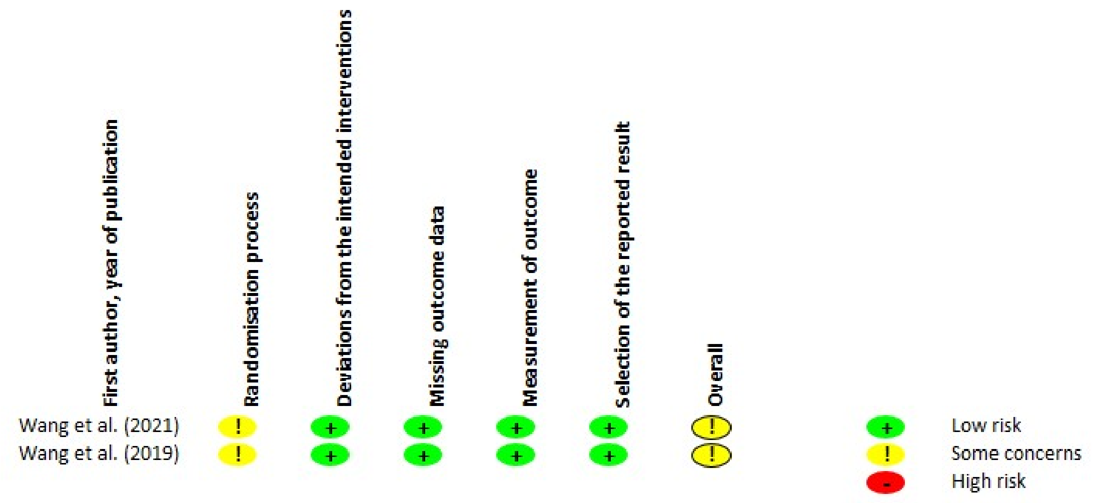

Figure S66. Risk of bias assessment of the studies included in the systematic review assessing resistance index (RI) and pulsatility index (PI) of uterine arteries [6,10] broken down to tools, shown in percentage

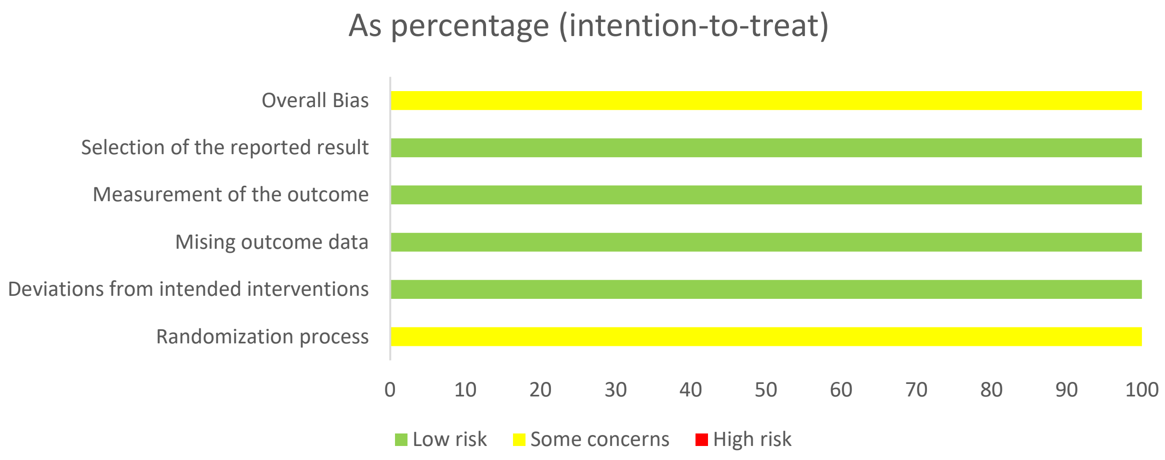

Figure S67. Risk of bias assessment of the studies included in the systematic review assessing vascular endothelial growth factor (VEGF) and/or integrin alpha vß3 [10,14] using the revised tool for assessing risk of bias in randomized trials (Rob 2)

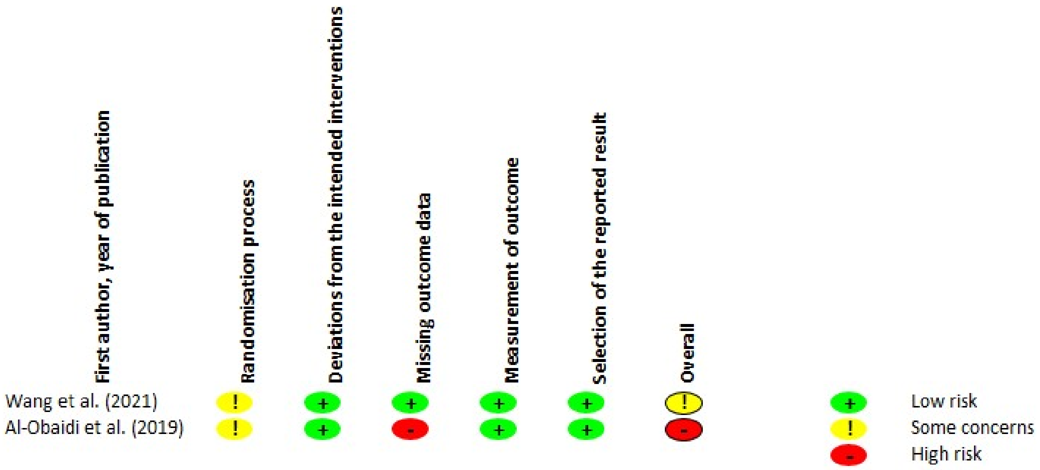

Figure S68. Risk of bias assessment of the studies included in the systematic review assessing vascular endothelial growth factor (VEGF) and/or integrin alpha vß3 [10,14] broken down to tools, shown in percentage

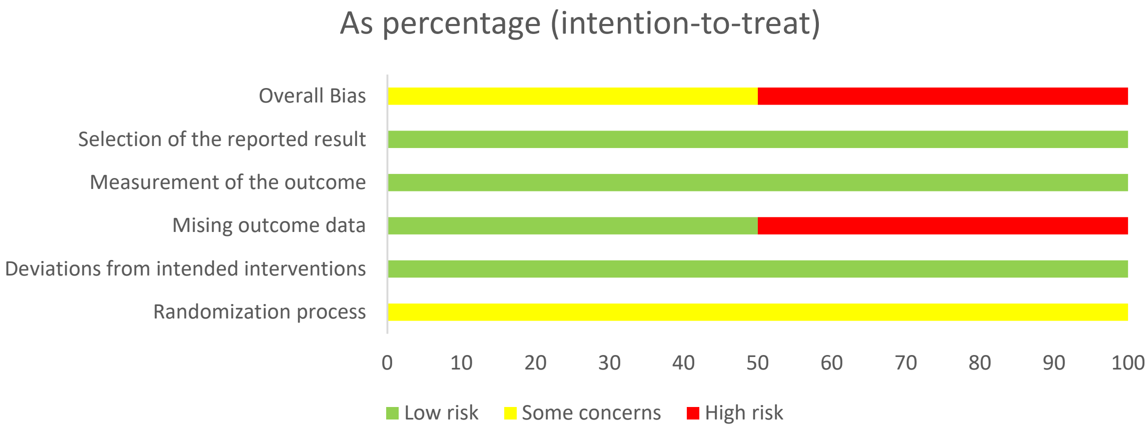

Figure S69. Risk of bias assessment of the studies included in the systematic review assessing resistance index (RI) and pulsatility index (PI) of subendometrial arteries [18] using the revised tool for assessing risk of bias in randomized trials (Rob 2)

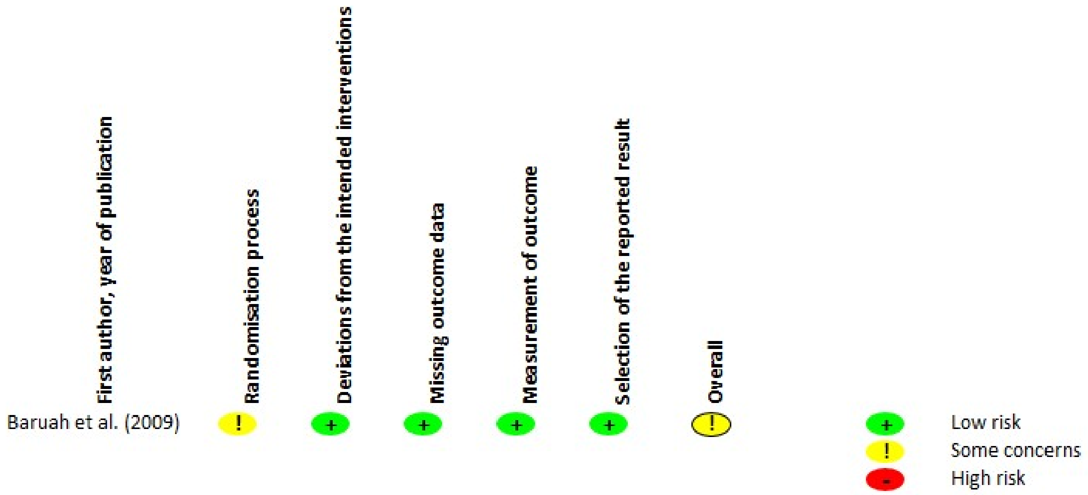

Figure S70. Risk of bias assessment of the studies included in the systematic review assessing resistance index (RI) and pulsatility index (PI) of subendometrial arteries [18] broken down to tools, shown in percentage

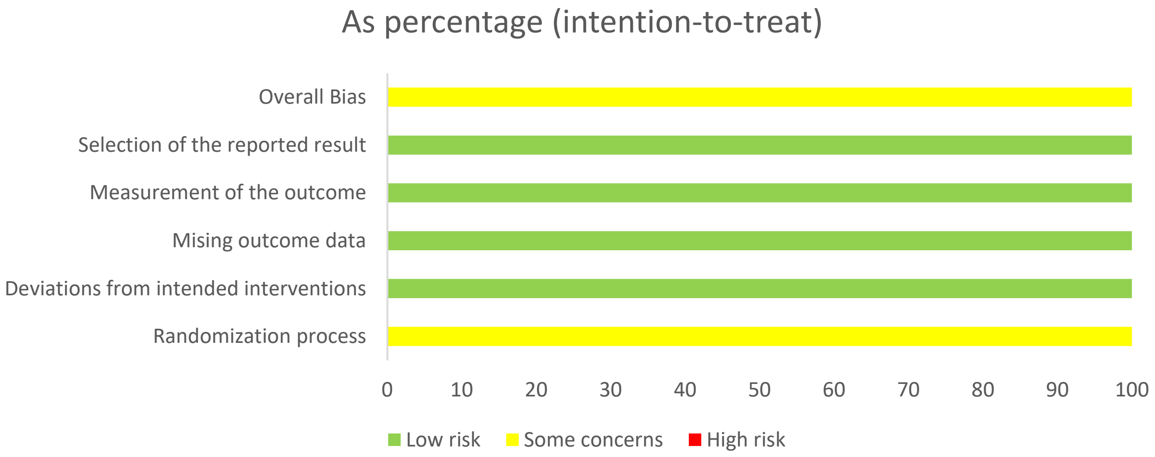

Supporting References

1. Page, M.A.-O.; McKenzie, J.E.; Bossuyt, P.M.; Boutron, I.; Hoffmann, T.C.; Mulrow, C.D.; Shamseer, L.; Tetzlaff, J.M.; Akl, E.A.; Brennan, S.E.; et al. The PRISMA 2020 statement: an updated guideline for reporting systematic reviews. **2021**, 372:n371, doi:10.1136/bmj.n71.
2. Kar, S. Clomiphene citrate or letrozole as first-line ovulation induction drug in infertile PCOS women: a prospective randomized trial. *Journal of human reproductive sciences* **2012**, *5*, 262-265, doi:10.4103/0974-1208.106338.
3. Zafar, T.; Asif, F.; Naurin, R.; Majeed, T.; Mahmood, Z. Comparing effectiveness of letrozole versus clomiphene citrate to evaluate the ovulation induction in patients with polycystic ovarian syndrome. *Pakistan journal of medical and health sciences* **2021**, *15*, 2685-2688, doi:10.53350/pjmhs2115102685.
4. Khakwani, M.; Parveen, R.; Yousaf, S.; Tareen, A.U. Efficacy of letrozole versus clomiphene citrate on ovulation induction in patients with polycystic ovarian syndrome. *Pakistan journal of medical sciences* **2022**, *38*, 1155-1158, doi:10.12669/pjms.38.5.5565.
5. Selim, M.F.; Borg, T.F. Letrozole and clomiphene citrate effect on endometrial and subendometrial vascularity in treating infertility in women with polycystic ovary syndrome. *Journal of Gynecologic Surgery* **2012**, *28*, 405-410, doi:10.1089/gyn.2012.0033.
6. Wang, L.; Wen, X.; Lv, S.; Zhao, J.; Yang, T.; Yang, X. Comparison of endometrial receptivity of clomiphene citrate versus letrozole in women with polycystic ovary syndrome: a randomized controlled study. *Gynecol Endocrinol* **2019**, *35*, 862-865, doi:10.1080/09513590.2019.1612358.
7. Najafi, P.Z.; Noghabi, S.P.; Afzali, N.; Mohammadzadeh, S. Comparing the effect of clomiphene citrate and letrozole on ovulation induction in infertile women with polycystic ovary syndrome. *J Pak Med Assoc* **2020**, *70*, 268-271, doi:10.5455/jpma.267607.
8. Hendawy, S.F.; Samaha, H.E.; Elkholy, M.F. Letrozole versus Clomiphene Citrate for Induction of Ovulation in Patients with Polycystic Ovarian Syndrome Undergoing Intrauterine Insemination. *Clin Med Insights Reprod Health* **2011**, *5*, 11-16, doi:10.4137/cmrh.S6598.
9. Mobusher, I. Comparison of the Efficacy of Letrozole and Clomiphene Citrate for Ovulation Induction in Infertile Women with Polycystic Ovary Syndrome. . *905 P J M H S* **2014**, *VOL. 8 NO.4*
10. Wang, L.; Lv, S.; Li, F.; Bai, E.; Yang, X. Letrozole Versus Clomiphene Citrate and Natural Cycle: Endometrial Receptivity During Implantation Window in Women With Polycystic Ovary Syndrome. **2021**.
11. Dehbashi, S., Dehbashi, Sa., Kazerooni, T., Robati, M., Alborzi, S., Ebrahim, M., Parsanezhad, Shadman, P.A. Comparison of the Effects of Letrozole and Clomiphene Citrate on Ovulation and Pregnancy Rate in Patients with Polycystic Ovary Syndrome. *Iranian Journal of Medical Sciences* **2009**, *34 No 1*.
12. Elsedeek, M.S.-E.-A.; Elmaghraby, H.A.H. Predictors and characteristics of letrozole induced ovulation in comparison with clomiphene induced ovulation in anovulatory PCOS women. *Middle East Fertility Society Journal* **2011**, *16*, 125-130, doi:10.1016/j.mefs.2010.11.004.
13. Atay, V.; Cam, C.; Muhcu, M.; Cam, M.; Karateke, A. Comparison of letrozole and clomiphene citrate in women with polycystic ovaries undergoing ovarian stimulation. *J Int Med Res* **2006**, *34*, 73-76, doi:10.1177/147323000603400109.
14. Al-Obaidi, M.T.; Ali, Z.H.; Saadi Wi, A.L.; Wasiti Ear, A.L.; Al-Aubaidy, H. Impact of letrozole versus clomiphene citrate on endometrial receptivity in Iraqi women with polycystic ovarian syndrome. *Journal of clinical pharmacy and therapeutics* **2019**.
15. Hussein, Z.; Al-Obaidi, M.T.; Al-Saadi, W.I.; Selman, M.O. Comparison of the effect of clomiphene citrate and letrozole on the endometrial parameters of PCOS women. *Journal of pharmaceutical sciences and research* **2017**, *9*, 2291-2295.

16. Ghahiri, A.; Mogharehabed, N.; Mamourian, M. Letrozole as the first-line treatment of infertile women with poly cystic ovarian syndrome (PCOS) compared with clomiphene citrate: A clinical trial. *Adv Biomed Res* **2016**, *5*, 6, doi:10.4103/2277-9175.175237.
17. Amer, S.A.; Smith, J.; Mahran, A.; Fox, P.; Fakis, A. Double-blind randomized controlled trial of letrozole versus clomiphene citrate in subfertile women with polycystic ovarian syndrome. *Hum Reprod* **2017**, *32*, 1631-1638, doi:10.1093/humrep/dex227.
18. Baruah, J.; Roy, K.K.; Rahman, S.M.; Kumar, S.; Sharma, J.B.; Karmakar, D. Endometrial effects of letrozole and clomiphene citrate in women with polycystic ovary syndrome using spiral artery Doppler. *Arch Gynecol Obstet* **2009**, *279*, 311-314, doi:10.1007/s00404-008-0714-4.
19. Bayar, U.; Basaran, M.; Kiran, S.; Coskun, A.; Gezer, S. Use of an aromatase inhibitor in patients with polycystic ovary syndrome: a prospective randomized trial. *Fertility and sterility* **2006**, *86*, 1447-1451, doi:10.1016/j.fertnstert.2006.04.026.
20. Roy, K.K.; Baruah, J.; Singla, S.; Sharma, J.B.; Singh, N.; Jain, S.K.; Goyal, M. A prospective randomized trial comparing the efficacy of Letrozole and Clomiphene citrate in induction of ovulation in polycystic ovarian syndrome. *J Hum Reprod Sci* **2012**, *5*, 20-25, doi:10.4103/0974-1208.97789.
21. Sakar, M.N.; Oglak, S.C. Letrozole is superior to clomiphene citrate in ovulation induction in patients with polycystic ovary syndrome. *Pakistan Journal of Medical Sciences* **2020**, *36*, 1460-1465, doi:10.12669/pjms.36.7.3345.
22. Sharief, M.; Nafee, N.R. Comparison of letrazole and clomiphene citrate in women with polycystic ovaries undergoing ovarian stimulation. *J Pak Med Assoc* **2015**, *65*, 1149-1152.
23. Al-Shaikh, S.F.M.H.; Al-Mukhatar, E.J.; Al-Zubaidy, A.A.; Al-Rubaie, B.J.U.; Al-Khuzaee, L. Use of clomiphene or letrozole for treating women with polycystic ovary syndrome related subfertility in Hilla city. *Middle East Fertility Society Journal* **2017**, *22*, 105-110, doi:10.1016/j.mefs.2016.12.003.
24. Ray, P.B.; Ray, A.; Chakraborti, P.S. Comparison of efficacy of letrozole and clomiphene citrate in ovulation induction in Indian women with polycystic ovarian syndrome. **2012**.
25. Bansal, S.; Goyal, M.; Sharma, C.; Shekhar, S. Letrozole versus clomiphene citrate for ovulation induction in anovulatory women with polycystic ovarian syndrome: A randomized controlled trial. *International Journal of Gynaecology and Obstetrics: the official organ of the International Federation of Gynaecology and Obstetrics* **2020**, *152*, 345-350, doi:10.1002/ijgo.13375.
26. Legro, R.S.; Brzyski, R.G.; Diamond, M.P.; Coutifaris, C.; Schlaff, W.D.; Casson, P.; Christman, G.M.; Huang, H.; Yan, Q.; Alvero, R.; et al. Letrozole versus clomiphene for infertility in the polycystic ovary syndrome. *N Engl J Med* **2014**, *371*, 119-129, doi:10.1056/NEJMoa1313517.
27. Schünemann, H.J.; Higgins, J.P.T.; Vist, G.E.; Glasziou, P.; Akl, E.A.; Skoetz, N.; Guyatt, G.H. Chapter 14: Completing 'summary of findings' tables and grading the certainty of the evidence. In Cochrane Handbook for Systematic Reviews of Interventions. Cochrane: 2022. **2022**.
